# Parameter estimation and identifiability analysis for a bivalent analyte model of monoclonal antibody-antigen binding

**DOI:** 10.1016/j.ab.2023.115263

**Published:** 2023-10-15

**Authors:** Kyle Nguyen, Kan Li, Kevin Flores, Georgia D. Tomaras, S. Moses Dennison, Janice M. McCarthy

**Affiliations:** a Biomathematics Graduate Program, North Carolina State University, Raleigh, 27607, NC, USA; b Center for Research in Scientific Computation, North Carolina State University, Raleigh, 27607, NC, USA; c Center for Human Systems Immunology, Duke University, Durham, 27701, NC, USA; d Department of Surgery, Duke University, Durham, 27710, NC, USA; e Department of Mathematics, North Carolina State University, Raleigh, 27607, NC, USA; f Department of Biostatistics and Bioinformatics, Duke University, Durham, 27710, NC, USA; g Department of Integrative Immunobiology, Duke University, Durham, 27710, NC, USA; h Department of Molecular Genetics and Microbiology, Duke University, Durham, 27710, NC, USA; i Duke Human Vaccine Institute, Duke University, Durham, 27710, NC, USA

**Keywords:** Bivalent analyte, Binding kinetics, Parameter identifiability, Surface plasmon resonance

## Abstract

Surface plasmon resonance (SPR) is an extensively used technique to characterize antigen-antibody interactions. Affinity measurements by SPR typically involve testing the binding of antigen in solution to monoclonal antibodies (mAbs) immobilized on a chip and fitting the kinetics data using 1:1 Langmuir binding model to derive rate constants. However, when it is necessary to immobilize antigens instead of the mAbs, a bivalent analyte (1:2) binding model is required for kinetics analysis. This model is lacking in data analysis packages associated with high throughput SPR instruments and the packages containing this model do not explore multiple local minima and parameter identifiability issues that are common in non-linear optimization. Therefore, we developed a method to use a system of ordinary differential equations for analyzing 1:2 binding kinetics data. Salient features of this method include a grid search on parameter initialization and a profile likelihood approach to determine parameter identifiability. Using this method we found a non-identifiable parameter in data set collected under the standard experimental design. A simulation-guided improved experimental design led to reliable estimation of all rate constants. The method and approach developed here for analyzing 1:2 binding kinetics data will be valuable for expeditious therapeutic antibody discovery research.

## Highlights

• An efficient workflow was developed to analyze bivalent analyte-ligand binding data.
• A grid search approach was used to estimate parameters with globally minimal standard error.
• We used profile likelihood to determine practically unidentifiable parameters.
• Simulated data was used to design experiment to overcome parameter non-identifiability.
• A HIV-1 antibody-antigen interaction was successfully analyzed using this workflow.

## Graphical abstract

**Figure:**
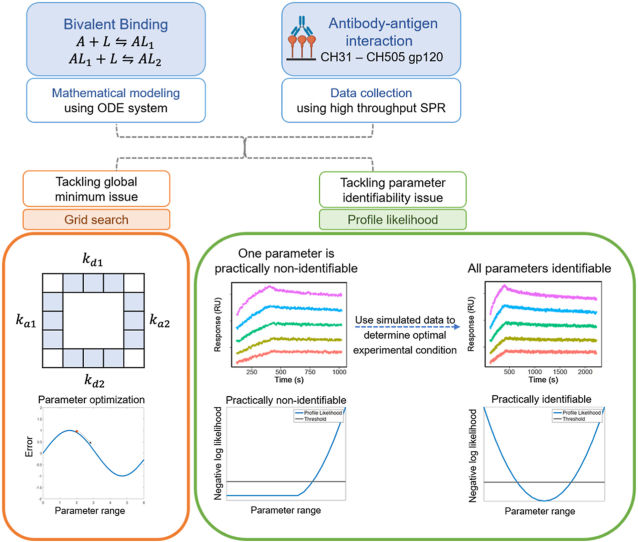

## 1 Introduction

A diverse range of antibodies can be elicited when the human immune system is exposed to a given pathogen. The binding affinities of monoclonal antibodies (mAbs) towards different antigens and domains within can be used to infer their domain and epitope specificity. Therefore, accurate modeling and determination of antibody-antigen binding affinities is crucial for understanding the mechanism of epitope recognition and how it relates to antibody function.

The label-free Surface Plasmon Resonance (SPR) platforms provide a powerful tool for determining binding affinities of antibodies [1]. Affinity measurements of antibody-antigen binding by SPR are usually carried out by immobilizing the bivalent antibodies (ligand) on the sensor surface and testing the binding of antigens (analyte) in solution. The SPR method is used to collect kinetics data by detecting changes in the resonance angle due to mass changes on the SPR chip surface during binding events [[2], [3], [4]]. Titrating the analyte using multiple concentrations and then globally analyzing the titration data to uniquely determine a single set of association and dissociation rate constants enhances the accuracy of affinity determination.

Typically, an SPR binding kinetics assay consists sequentially of ligand immobilization, baseline, analyte association and analyte dissociation steps, followed by an optional regeneration step. A solution containing the analyte molecule in buffer is interacting with the sensor chip during the association step and only the corresponding buffer is interacting with the sensor chip during the dissociation step. If the analyte is being titrated at multiple concentrations, typically from low to high, *i.e.*, during a kinetics titration [5], the baseline, association and dissociation steps will be repeated for each concentration.

Whether to implement the regeneration step depends on the ligand. The ligand can be permanently immobilized though procedures such as amine-coupling or streptavidin capturing, or non-permanently captured using immobilized reagents that show strong affinity to the ligand. During the regeneration step, a solution of extreme pH or high salt concentration is typically used. If the ligand is permanently immobilized, regeneration can rapidly dissociate the analyte from the immobilized ligand. If the ligand is non-permanently captured, regeneration can dissociate the analyte-ligand pairs from the ligand-capturing molecules, enabling re-capturing of the ligand before the next titration cycle. However, permanently immobilized ligands are often sensitive to the regeneration buffer used; the re-capturing of ligand in every cycle could also lead to longer experiment time and higher reagent consumption. In these cases, the kinetics titrations will be performed without regeneration [5], and therefore the SPR chip is not completely free of bound analyte when the next cycle starts.

There are multiple models to consider when analyzing SPR binding kinetics data. How to identify the appropriate model, *i.e.*, model identification, has been explored previously [6,7], for example by Tiwari et al. [7]. Briefly, the simplest model for fitting of SPR titration data of antibody-antigen interactions is the 1:1 Langmuir model of monovalent binding [[8], [9], [10], [11]]. This model is suited when a mAb is immobilized and a single binding site antigen is used as an analyte. More complex binding interactions require other binding models [[12], [13], [14]], such as a bivalent analyte model, bivalent ligand model, heterogenous ligand model or a two-step conformational change model. It is important to choose a biochemically relevant model to accurately reflect the underlying kinetic process of the molecular interaction. One should not choose a complex kinetics model simply because it fits the data better.

When studying interactions using complex models, instead of solving the linear differential equations, the kinetics parameters can be obtained by fitting the SPR data to a system of non-linear ordinary differential equations (ODEs), using a numeric integrator to solve the system and an optimization algorithm that compares the model's predictions to the data and finds the “best” parameters by minimizing the error in predicted outcome versus observed.

**Fig. 1:**
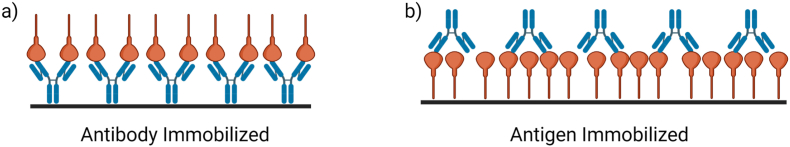
**The orientation of binding impacts kinetics characteristics.** Cartoon illustration of binding modes when either antibody or antigen is immobilized. The scenario of antibody being immobilized is shown in a), where 1:1 binding interaction can be assumed. The scenario of antigen being immobilized is shown in b), where bivalent model is needed.

Among the multivalent binding models, the bivalent analyte model can be particularly useful in evaluation of antibody binding breadth for antigen variants. For example, a panel of antigens can be immobilized, and a single antibody can be used as the analyte to determine the binding affinities for these antigens. Due to the bivalency of the antibody, the bivalent analyte model needs to be implemented. In SPR, the response is proportional to the amount of analyte bound to the surface. When the antibody is immobilized, response increases for each binding site bound to analyte, so that there is a 1:1 interaction between each bound analyte and binding site on the ligand. In contrast, when bivalent antibody is used as the *analyte*, the binding of the second arm of the antibody to an immobilized ligand does not result in further change of response, so that there can be a decrease of free ligand molecules with no change in response. Fig. 1 illustrates how the binding modes differ depending on the whether the antibody or antigen is immobilized.

Mathematical models for bivalent analyte binding kinetics data have previously been developed and studied [6,7,15], however, the existing tools for analyzing data using a bivalent analyte model have several limitations. The commercial software programs that exist and have been used to estimate the association and dissociation rate constants of bivalent analyte interactions [[16], [17], [18], [19], [20]] are designed for low-throughput instruments and mostly for regenerative titration cycles. Further, the existing models do not address two important issues common in non-linear optimization: local minima and parameter identifiablity. These latter limitations can result in the reporting of erroneous parameter estimates.

**Fig. 2:**
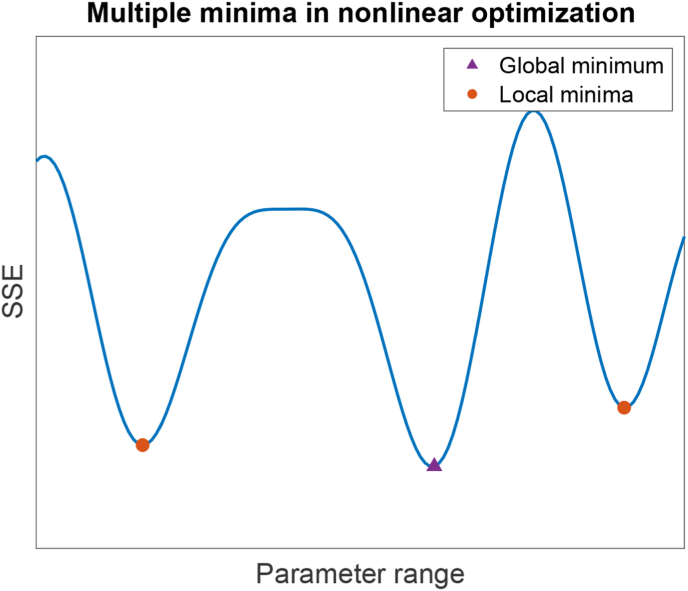
**Illustrative example of a multiple minima problem.** Illustrative example of a multiple minima problem that is common in nonlinear optimization is shown. The blue curve represents the sum of squares error (*SSE*) as a function of different values of a parameter. The orange dots are local minima and the purple triangle is the global minimum.

The local minima problem concerns all parameters. If parameter optimization can be compared to searching along a very “bumpy” curve consisted of multiple “valleys” (minima), with one lowest “valley” (global minimum), depending on the parameter initializations, the algorithm might search in small steps to find the global minimum, or get “stuck” in a local minimum. As an example, Fig. 2 illustrates a hypothetical case where the error (sum of squared error) is a function of a single parameter value.

In contrast, non-identifiability concerns one or more specific parameters. The specific parameter(s) may not be reliably estimated when different values of the parameter(s) lead to the same (or numerically similar) values in the error function. Identifiability issues can arise when a model has many parameters compared to observed data or when there are unobserved states. Mathematical methods for parameter identifiability analysis have been developed, described, and improved upon in the literature [21,22] and have been applied in many biomathematical models, for example in epidemiology [[23], [24], [25]] and oncology [26,27]. However, the identifiability of the parameters for bivalent analyte binding model is yet to be investigated.

In this work, we aim to optimize the performance of a bivalent analyte model by tackling the local minima and non-identifiability issues. For the problem of local minima, we performed parameter estimation at multiple sets of initial guesses that cover wide ranges of numerical values, and record the optimized parameters with the lowest error. This approach ensures the parameter search reaches the global minimum. For the problem of parameter non-identifiability, we went beyond the usual mathematical analysis and used simulation to guide experimental design. This computational step is especially important for improving resource and time efficiency during the data collection step while ensuring that there is sufficient information in the data to fit the model. We optimize the model using the binding kinetics data of a broadly neutralizing HIV-1 mAb binding to HIV-1 envelope glycoprotein gp120, demonstrating that our approach is directly applicable to study antibody-antigen interactions.

## 2 Materials and methods

### 2.1 SPR kinetics data collection

The SPR binding kinetics measurements of HIV-1 mAb was done on a Carterra LSA platform using HC200 M sensor chips (Carterra) at 25^*o*^C, with a data acquisition rate of ∼ 0.5 Hz. Two microfluidic modules used to deliver liquids onto the sensor chip were a 96-channel print-head (96 PH) and a single flow cell (SFC).

The chip was first activated by 100 mM N-Hydroxysuccinimide (NHS) and 400 mM 1-Ethyl-3-(3-dimethylaminopropyl) carbodiimide hydrochloride (EDC) (Cytiva, mixed 1:1:1 with 0.1 M 2-(N-morpholino) ethanesulfonic acid (MES) buffer at pH 5.5) for 600 seconds (s), followed by direct immobilization of CH505 transmitted founder (T/F) gp120 [28] (in 10 mM Sodium Acetate at pH 4.5) at multiple concentrations for 600 s using the 96 PH. Unreactive esters were then quenched with a 600 s injection of 1 M ethanolamine-HCl at pH 8.5. The running buffer was 10 mM MES buffer at pH 5.5 with 0.01% Tween-20, and each concentration of CH505 T/F gp120 was immobilized onto up to 24 separate spots of the same chip. Unless specified above, the steps were done using the SFC. CH505 T/F gp120 was produced by Duke Human Vaccine Institute Protein Production Facility as described earlier [28] and further purified by size exclusion chromatography for monomeric gp120.

A two-fold dilution series of the CH31, a HIV-1 mAb with CD4-binding site specificity [29], was prepared in 1x HBSTE (10 mM HEPES pH 7.4, 150 mM NaCl, 3 mM EDTA and 0.01% Tween-20) buffer. The highest concentration was 150 μg/ml (1.0 μM). CH31 mAb at different concentrations was then injected using SFC onto the chip surface from the lowest to the highest concentration, including 8 injections of buffer before the lowest non-zero concentration for signal stabilization. For each concentration, the time-length of data collection for baseline and association was 120 s and 300 s, respectively; the standard time-length for dissociation was 600 s and the extended time-length for dissociation was 1800 s. If the chip surface was regenerated at the end of dissociation, double pulses of 10 mM Glycine HCl at pH 2.0 was used for regeneration, with 30 s per pulse. For all assays the running buffer for titration was 1X HBSTE.

The titration data collected were first pre-processed in the Kinetics (Carterra) software, including reference subtraction using spots with no immobilized biomolecules, buffer subtraction using the last zero-concentration cycle and data smoothing. The data were then imported into Excel from the Kinetics package. Spots that show sensorgrams with good dose dependence and least amount of noise were down-selected for bivalent model analysis.

### 2.2 Mathematical modeling of bivalent analyte binding

The simplest model for binding kinetics is the 1:1 Langmuir model, described by the following pseudo-reaction:

(1) $$A+L\underset{k_{d}}{\overset{k_{a}}{⇌}}AL$$

where *A* is the analyte in the solution, and *L* is the immobilized ligand on the sensor. This model assumes that one analyte only binds with one ligand to form the complex *AL* with an association rate constant *k*_*a*_. The complex *AL* can dissociate into *A* and *L* with a dissociation rate constant *k*_*d*_.

Bivalent analyte binding kinetics can be represented using the following two reversible pseudo reactions:

(2) $$A+L\underset{k_{d1}}{\overset{k_{a1}}{⇌}}AL_{1}$$

(3) $$AL_{1}+L\underset{k_{d2}}{\overset{k_{a2}}{⇌}}AL_{2}$$

where *A* is the bivalent analyte in the solution, and *L* is the immobilized ligand on the sensor. The complex *AL*_1_ is formed when a ligand binds with one arm of an analyte at the association rate constant *k*_*a*1_. *AL*_1_ can revert back to *A* and *L* at the rate constant *k*_*d*1_. In the second reaction, the remaining arm of *AL*_1_ can associate to and dissociate from another ligand at the association and dissociation rate constants, *k*_*a*2_ and *k*_*d*2_, respectively. These processes are further illustrated in Fig. 5a. It is important to note that we are unable to observe the difference between [*AL*_1_] and [*AL*_2_].

**Fig. 3:**
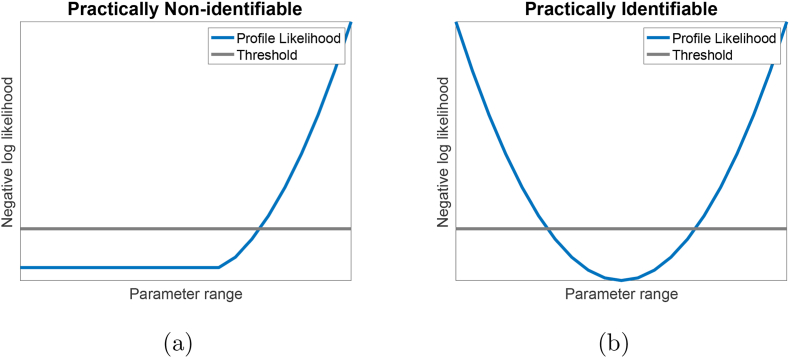
**Illustrative examples of practically non-identifiable and practically identifiable parameter.** Illustrative example profile likelihood for: **(a)** a practically non-identifiable parameter, and **(b)** a practically identifiable parameter. In (a), comparing to the threshold (**gray** line), the profile likelihood (**blue** curve) for a practically non-identifiable parameter is flat on one side and manifests an *L*-shape curve. On the other hand, in (b), a practically identifiable parameter has a bowl-shape profile likelihood with a clear minimum.

**Fig. 4:**
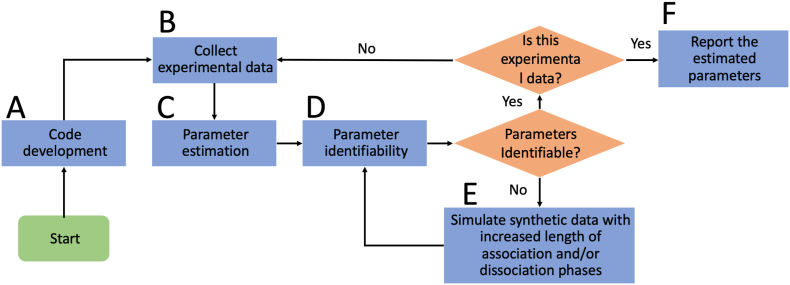
**Schematic for the experimental procedure.** Schematic depicting the step-by-step process for reliable determination of the kinetics parameters for the bivalent analyte model. (A) First, we start with the code development and strategy for parameter estimation for data collected in high throughput fashion. (B) Next, we collect experimental data. (C) We then obtain estimates of the parameters using non-linear optimization. (D) After obtaining the estimates, we examine the identifiability of the parameters. (E) If a parameter is found to be non-identifiable, we perform simulations to find the optimal data collection scheme in which that parameter is identifiable. After finding the optimal scheme, we restart the process from step (B) and confirm that the parameters are identifiable. (F) Finally, we report the estimated parameters.

**Fig. 5:**
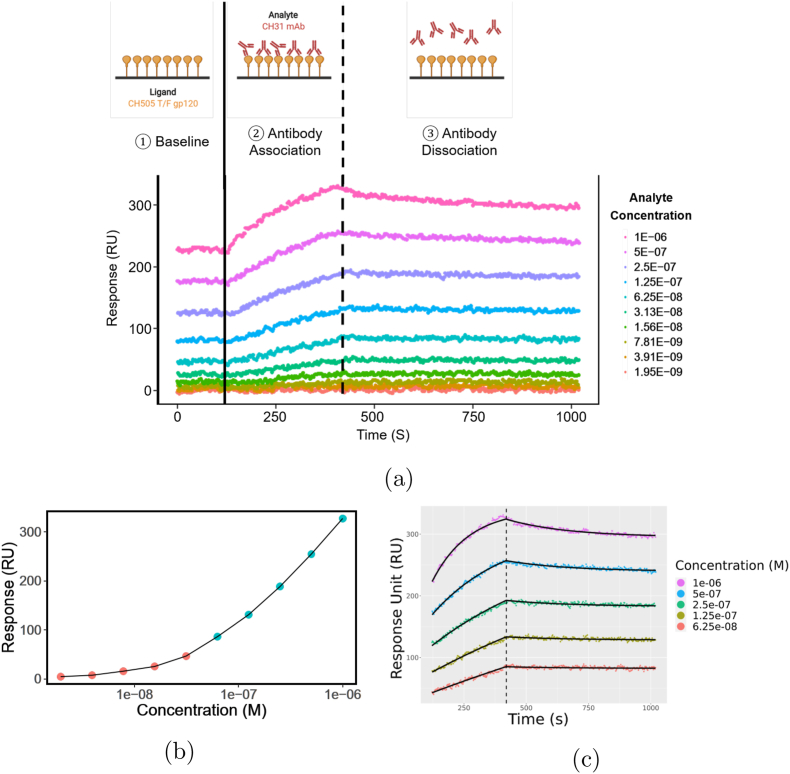
**Bivalent analyte association and dissociation processes with a representative sensorgram. (a)** An example sensorgram of a bivalent analyte CH31 mAb binding to a transmitted/founder CH505 HIV-1 envelope glycoprotein gp120 antigen (ligand) is shown. The 10 **dotted** curves correspond to 10 different concentrations of CH31 mAb as analyte, with the concentration values shown in the legend. The left vertical **black solid** line separates the baseline and association, while the right vertical **black dashed** line separates the association and dissociation steps. The cartoon above the sensorgram illustrates each step in a titration cycle: (1) During the baseline phase, the response on the sensor is stabilized. (2) During the association phase, analyte solution that contains CH31 mAb is flowed over. The analytes start associating to the ligands (CH505 T/F gp120) on the sensor. This results in an increase in response. During the dissociation phase, buffer solution with no analyte is flowed allowing the analytes to begin dissociating from the ligands, resulting in decreasing response. **(b)** A plot of binding response averaged at the end of association step is shown as a function of concentration of CH31 mAb. The concentrations in **green** in panel **(b)** are chosen for fitting kinetic constants. **(c)** Bivalent analyte model fitted sensorgrams of CH31 mAb binding to CH505 T/F gp120 are shown.

We separately modeled the association phase and the dissociation phase as two sub-models as described in Section 2, 2.2.1.2.2, respectively. It should be noted that both analyte association and analyte dissociation occur during the association phase. However, to avoid confusion, we will adhere to the SPR convention and use the term “association phase”.

#### 2.2.1 Association phase model

For the association phase, one can derive a bivalent analyte model that consists of ordinary differential equations (ODEs) from the reactions (2) and (3). In this study, we employ a model that is used by commercial software, ForteBio [30] and Biacore [31]. The model is described by the following equations:

(4) $$\frac{\text{d}\left[L\right]}{\text{d}t}=-\left(2k_{a1}\left[A_{m}\right]\left[L\right]-k_{d1}\left[AL_{1}\right]\right)-\left(k_{a2}\left[AL_{1}\right]\left[L\right]-2k_{d2}\left[AL_{2}\right]\right)$$

(5) $$\frac{\text{d}\left[AL_{1}\right]}{\text{d}t}=\left(2k_{a1}\left[A_{m}\right]\left[L\right]-k_{d1}\left[AL_{1}\right]\right)-\left(k_{a2}\left[AL_{1}\right]\left[L\right]-2k_{d2}\left[AL_{2}\right]\right)$$

(6) $$\frac{\text{d}\left[AL_{2}\right]}{\text{d}t}=\left(k_{a2}\left[AL_{1}\right]\left[L\right]-2k_{d2}\left[AL_{2}\right]\right)$$

where the [*A*_*m*_] represents the analyte concentration in the solution and is assumed constant as analyte is continually supplied. [*L*], [*AL*_1_], and [*AL*_2_] represent the concentrations of free ligand, analyte-ligand complex, and analyte-two-ligand complex, respectively. The first two terms of each Eqs. (4), (5) are derived from Eq. (2). The concentration of free ligand [*L*] decreases due to the formation of [*AL*_1_] when a free ligand binds with one of an analyte. The factor of two in the first term of Eq. (4) accounts for the fact that the free ligand can bind at either arm of the analyte. On the other hand, the concentrations of free ligand increases proportionally to the decomposition of *AL*_1_. In the first reaction (Eq. (2)), the change in concentration of analyte-ligand, [*AL*_1_], is opposite to the change of [*L*]. As the concentration of free ligand increases or decreases, the concentration of analyte-ligand complex decreases or increases.

This model assumes a sequential two-step process, where the binding and unbinding have to occur in order. This means *AL*_2_ cannot be formed before the formation of *AL*_1_. Similarly, *AL*_1_ cannot dissociate before the dissociation of *AL*_2_. These assumptions explain the remaining terms in Eqs. (4), (5), (6). The concentration of analyte-two-ligand complex ([*AL*_2_]) increases proportionally to both concentrations of the free ligand and the analyte-ligand. The analyte-two-ligand complex decompose into an analyte-ligand complex and a ligand. Note that there are again multiple factors of two in these terms to account for the fact that the bound analyte can unbind with either of its arms.

In an experiment where the surfaces were regenerated between injection of different concentration of analyte, we can set ${\left[AL_{1}\right]}_{0}={\left[AL_{2}\right]}_{0}=0$. In experiments without regeneration, the concentrations ${\left[AL_{1}\right]}_{0}$ and ${\left[AL_{2}\right]}_{0}$ are unknown. To address this, we extrapolate back in time by estimating an initial time adjustment, t*, and compute the initial time *t*_0_ for each concentration as follows:

(7) $$t_{0}=t_{0}^{*}-t^{*}$$

where $t_{0}^{*}$ is the initial starting time of the experiment. We then can assume that at this adjusted time *t*_0_, ${\left[AL_{1}\right]}_{0}={\left[AL_{2}\right]}_{0}=0$.

The initial amount of free ligand [*L*]_0_ is also unknown. We fit this as a parameter for each concentration.

#### 2.2.2 Dissociation phase model

In this model, we assume that the association rate constants, *k*_*a*1_ and *k*_*a*2_, are negligible and set them to zero. This means there is no rebinding of the analyte to the ligand at any given ligand density. Therefore, the reaction equations for the dissociation phase take the form of two pseudo decomposition reactions:

(8) $$AL_{1}\overset{k_{d1}}{\to }A+L$$

(9) $$AL_{2}\overset{k_{d2}}{\to }AL_{1}+L$$

Corazza et al. [14] also assumed strict decay of the bound species, but used a sum of exponential model with two parameters, *k*_*d*1_ and *k*_*d*2_. Here, rather than using a phenomenological model, we derived a mechanistic model that consists of the following ODEs:

(10) $$\frac{\text{d}\left[L\right]}{\text{d}t}=k_{d1}\left[AL_{1}\right]+2k_{d2}\left[AL_{2}\right]$$

(11) $$\frac{\text{d}\left[AL_{1}\right]}{\text{d}t}=-k_{d1}\left[AL_{1}\right]+2k_{d2}\left[AL_{2}\right]$$

(12) $$\frac{\text{d}\left[AL_{2}\right]}{\text{d}t}=-2k_{d2}\left[AL_{2}\right]$$

As previously stated in Section 2.2.1, the model follows the assumptions of a two-step process. However, unlike the association phase, during the dissociation phase, we assume the concentration of the analyte-two-ligand complex only decreases over time as *AL*_2_ decomposes into *AL*_1_ and *L*. Each analyte-single-ligand complex, *AL*_1_, is then decomposed into an analyte, *A*, and a ligand, *L*. By using a system of ODEs for the dissociation phase, we make the assumption that *AL*_2_ cannot directly decompose into an *A* and two *L* without decomposing into an *AL*_1_ and an *L* first. Further, we assume that *AL*_1_ cannot rebind to form *AL*_2_. Combining Eqs. (4), (5), (6) for association model with Eqs. (10), (11), (12), we have the bivalent analyte model.

### 2.3 Parameter estimation

Unlike the 1:1 Langmuir model, the bivalent analyte model is comprised of nonlinear ODEs, and analytical solutions do not exist. Therefore, we solve for the approximate solutions by integrating the ODEs numerically. In this work, we use the function ode from an R package called deSolve [32] to numerically approximate the solution of the model. Additionally, we use the nonlinear least squares function, nls.lm, from the package minpack [33], to fit the data using the Levenberg-Marquardt (LM) algorithm [34]. This algorithm is also used in the commercial software Biacore [31]. The LM algorithm estimates the parameters by minimizing the sum of squared error (*SSE*):

(13) $$SSE\left(p\right)=\overset{M}{\underset{j=1}{\sum }}\overset{N_{j}}{\underset{i=1}{\sum }}{\left[\hat{RU}\left(p,t_{i},{\left[A_{m}\right]}_{j}\right)-RU_{i,j}^{o}\right]}^{2},$$

where $\hat{RU}\left(p,t_{i},{\left[A_{m}\right]}_{j}\right)$ and $RU_{i,j}^{o}$, respectively, are model output response unit (RU) and observed response unit in the data at the *i*th time for the *j*th analyte concentration. *N*_*j*_ is the total number of data points observed for the *j*th concentration and *M* is the total number of concentrations being used during fitting. The vector **p** represents a vector of parameters to be estimated. To improve confidence in parameter estimation of the rate constants (e.g., *k*_*a*1_, *k*_*d*1_, *k*_*a*2_, and *k*_*d*2_), we fit all concentrations simultaneously. In this work, 5 concentrations corresponding to the linear range of the titration dose response curve are selected, *i.e.*, *M* = 5. This is referred to as a ‘global fit’ and has been shown to yield more robust and reliable results [30]. In addition to the global parameters, each analyte concentration also has a set of local parameters, *R*max_*j*_ and $t_{j}^{*}$, with *j* = 1, …, 5. *R*max's are the responses associated with maximum analyte bound to the surface, which is proportional to the maximum free ligand concentration on the sensor, [*L*]_0_. Because we are using non-regenerative titration data, we also need to fit an initial time adjustment, $t_{j}^{*}$, for each concentration.

(14) $$\hat{RU}\left(p,t_{i},{\left[A_{m}\right]}_{j}\right)={\left[AL_{1}\right]}_{i,j}+{\left[AL_{2}\right]}_{i,j}$$

We estimated 14 parameters for each data set: 4 global parameters (*k*_*a*1_, *k*_*d*1_, *k*_*a*2_, and *k*_*d*2_) and 10 local parameters ([*L*]_01, *…*,05_ and $t_{1,\ldots ,5}^{*}$). The summary details of all parameters for each model are shown in Table 1. We used a constrained optimization where the lower bound of all parameters was zero.

**Table 1:** Summary of parameters for the bivalent analyte model.

Parameter	Description	Unit	Fit
*k*_*a*1_	First association rate constant	*M*^−1^*s*^−1^	Global
*k*_*d*1_	First dissociation rate constant	*s*^−1^	Global
*k*_*a*2_	Second association rate constant	*RU*^−1^*s*^−1^	Global
*k*_*d*2_	Second dissociation rate constant	*s*^−1^	Global
*R*max	Response associated with the maximum analyte bound to ligand; proportional to [*L*]_0_	RU	Local
t*	Initial time adjustment for non-regenerative titration	*s*	Local

### 2.4 Identifiability analysis using the profile likelihood

One of the methods for identifiability analysis is the profile likelihood-based confidence intervals [21]. The profile likelihood creates a profile for each parameter across a reasonable range of values. Given the data, the negative log likelihood function is defined as:

(15) $$NLL\left(p\right)=\frac{N}{2}\ln\left(2\pi \right)+\frac{1}{2}\frac{SSE\left(p\right)}{\sigma^{2}}$$

where *NLL*(**p**) is the negative likelihood while **p** is the vector of model parameters. *N* is the number of measurements in the data. In addition, *SSE*(**p**) is computed using Eq. (13). *σ* is the measurement error. We assume that *σ* is known, is the same for all measurements, and can be approximated using residual errors. Furthermore, the minimum of the likelihood is independent of the constant number, $\frac{N}{2}\ln\left(2\pi \right)$. Therefore, minimizing the negative likelihood function *NLL* is equivalent to minimizing the function *SSE*. For each parameter *p*_*j*_, the profile likelihood *PLL*_*j*_ is computed using the following function [27]:

(16) $${PLL}_{j}\left(p\right)=\underset{\left\{p|p_{j}=c\right\}}{\text{min}}NLL\left(p\right)$$

where *c* is a fixed value for *p*_*j*_ within a predefined reasonable range. Simply put, to compute a profile likelihood for parameter *p*_*j*_, we fix *p*_*j*_ across a range of values with [min(*p*_*j*_), max(*p*_*j*_)]. Then, we perform parameter estimation as described in Section 2.3 for all parameters except *p*_*j*_ for each fixed value of *p*_*j*_. Finally, we compute *NLL* using the estimated parameters (with fixed *p*_*j*_). The computed *NLL*'s across all fixed values of *p*_*j*_ form a profile likelihood for *p*_*j*_. The pseudocode to compute profile likelihood is described in Algorithm C.1.

In Fig. 3a, we illustrate an example of a practically non-identifiable parameter, where the profile is flat on one side. On the other hand, when the profile likelihood is nonflat on both sides, as shown in Fig. 3b, the parameter is practically identifiable. To determine the flatness of the profile, we need to compute the flatness threshold. The threshold is computed as follows [27]:

(17) $$\text{Threshold}=\text{min }NLL\left(p\right)+\frac{\Delta_{\alpha }}{2},$$

where Δ_*α*_ = *χ*^2^(*α*, *df*), is the *α*-quantile for the *χ*^2^-distribution. Since we are computing the upper 95% confidence threshold, we choose *α* = 0.95. According to Raue et al., the degree of freedom, *df*, should be either 1 or *#p*, with *#p* being the number of parameters to be estimated [21]. To compute the threshold for each individual parameter, the degrees of freedom must be *df* = 1 [21]. We compute a joint threshold for all parameters, so we choose *df* = *#p* [21].

### 2.5 Iterative process of parameter optimization

In order to efficiently use time and materials for model development, we used an iterative process that combined the power of computation and experimental data collection. After the initial mathematical model building for bivalent analyte binding, we utilized data simulation to help fine-tune the experimental conditions that are both practical and provide sufficient information for reliable parameter estimations.

A schematic illustration of the model building and parameter optimization process is shown in Fig. 4. The initial model refinement and code development is followed by the first round of experimental data collection. Then parameter estimation and parameter identifiability analysis using the log profile likelihood [21] is carried out using the experimental data. If a parameter is found to be practically non-identifiable, simulated data is used to find a new experimental data collection scheme that would enable reliable parameter estimation. Then new experimental data collection will either validate the new scheme or lead to another round of optimization. The iterative process will end when all parameters are identifiable using the latest data collection scheme.

## 3 Results

### 3.1 Broadly HIV-1 neutralizing mAb CH31–CH505 gp120 binding data for code development

We implemented an iterative process of code development and experimental data collection and subsequent processing as outlined in Fig. 4. Experimental data was collected by performing titrations of a broadly neutralizing HIV-1 mAb CH31 [29] as the analyte binding to CH505 gp120 [28] antigen amine-coupled on a SPR chip, in a non-regenerative fashion. Among all replicates, 14 best replicates were selected for the purpose of model development. An example titration sensorgram after baseline correction is shown in Fig. 5a. Based on the dose response curve (Fig. 5b), we selected the data for the highest 5 concentrations in the titration series (molar concentration: 6.25 × 10^−8^, 1.25 × 10^−7^, 2.5 × 10^−7^, 5 × 10^−7^, and 10^−6^ M) for kinetics parameter estimation as these 5 concentrations best represent the linear range of the dose response.

### 3.2 A parameter grid search approach to address the local minima problem

Because the LM algorithm is a local optimization algorithm, it is prone to getting stuck in a local minimum when solving non-linear problems and outcomes can depend heavily on the initial starting values of parameters. Therefore, for a robust fit, we performed parameter estimation at multiple sets of initial guesses. For each kinetic parameter (*k*_*a*1_, *k*_*d*1_, *k*_*a*2_, and *k*_*d*2_), we implemented a grid search approach, where we examined all possible combinations of 3 different initial values for each of the four kinetics parameters, while using the same initial values for non-kinetic parameters. This results in 3^4^ or 81 different sets of initial guesses. After running parameter estimation at different initial guesses, we recorded the optimized parameters with the lowest error. Although this approach is computationally expensive, it ensures that recorded optimized parameters give the lowest possible SSE, reducing the chance that the estimated parameters are not optimal. In Table 2, we show a representation of the grid search outcome for the initial guesses for kinetics parameters for the first dataset of the bivalent analyte model. With “wrong” initial guesses, the algorithm could fail to achieve the global minimum error. For example, with $k_{a1}^{\left(0\right)}=10^{2}\;M^{-1}s^{-1}$, $k_{d1}^{\left(0\right)}=10^{-3}\;s^{-1}$, $k_{a2}^{\left(0\right)}=10^{-5}\;RU^{-1}s^{-1}$, and $k_{d2}^{\left(0\right)}=10^{-5}\;s^{-1}$ the LM algorithm is only able to converge locally. In contrast, if we change the initial guess $k_{a2}^{\left(0\right)}$ to 10^−4^ *RU*^−1^*s*^−1^, the algorithm is able to reach the global minimum error. Therefore, when using a local optimization algorithm such as the LM algorithm, it is crucial to test the results at multiple initial guesses to ensure a robust fitting result.

**Table 2:**
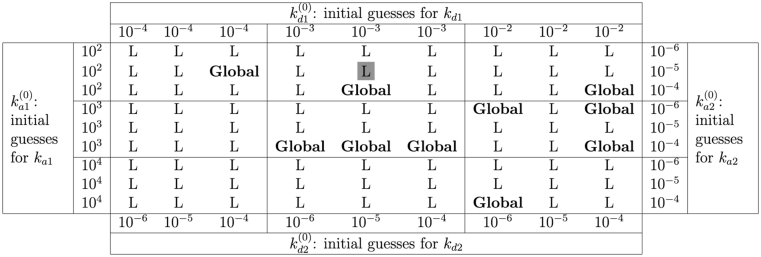
**Grid search results table for initial guesses.** Table for initial guesses grid search results for a representative dataset for the bivalent analyte model with standard length of dissociation. The outcomes are divided into two categories: L, and **Global**. Given a set of initial guesses, L stands for local minimum, where the LM algorithm gets stuck at the local minimum. In contrast, when the LM algorithm reaches the global minimum, we denote the result as **Global**. An example to read the table: at the gray-boxed L, with the initial guesses $\left(k_{a1}^{\left(0\right)},k_{d1}^{\left(0\right)},k_{a2}^{\left(0\right)},k_{d2}^{\left(0\right)}\right)=\left(10^{2},10^{-3},10^{-5},10^{-5}\right)$, the LM algorithm converges to a local minimum.

### 3.3 Kinetics parameters are not reliably estimated with standard length of dissociation phase

**Fig. A.1:**
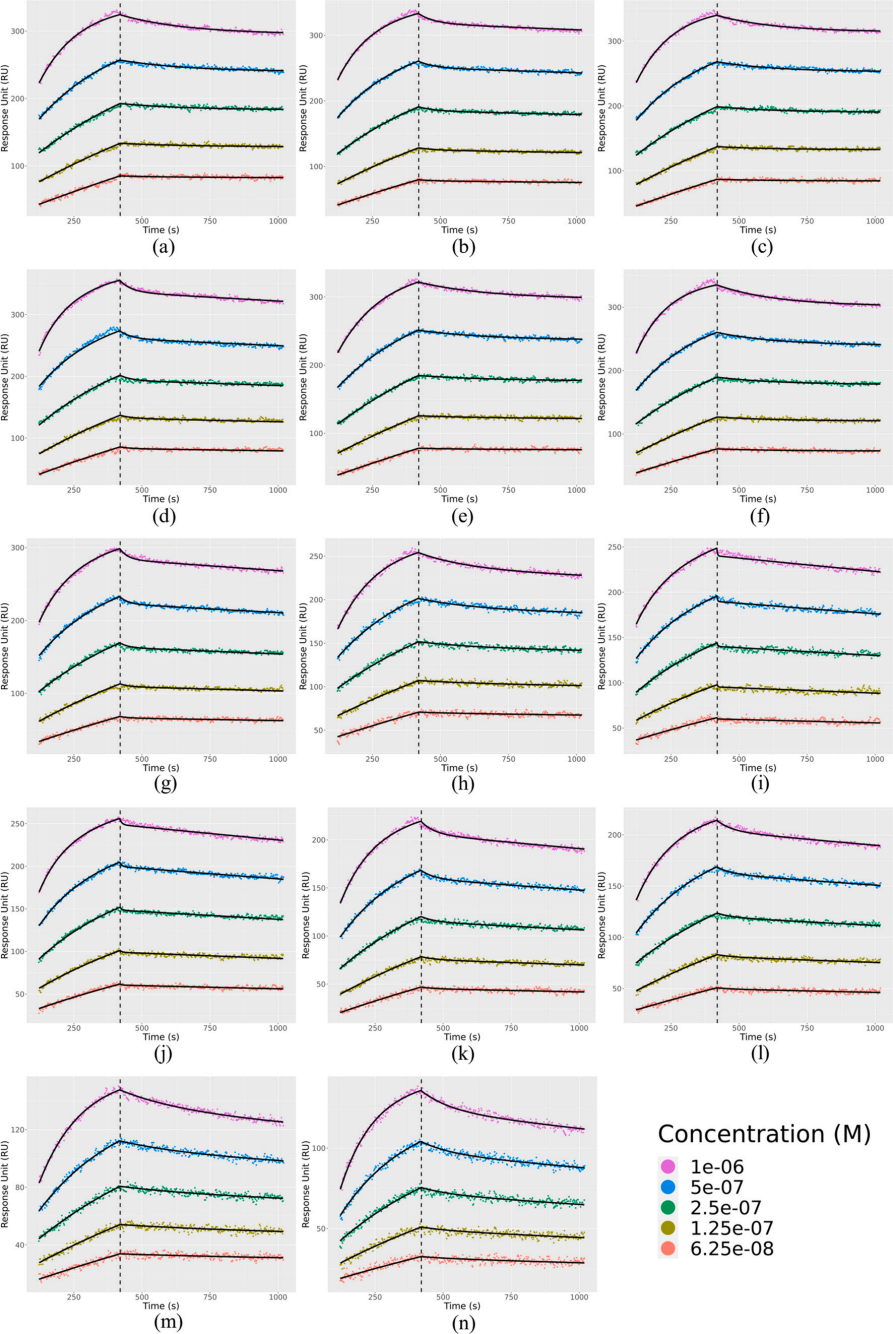
**Bivalent analyte model fitting results for the standard length of dissociation.** Model fitting results using the bivalent analyte model for: (a)–(n) data sets 1–14 with standard length of dissociation.

In this section, we present the results of fitting the bivalent analyte model using the standard length of dissociation phase, i.e., 600 s. The fitted sensorgram for one example data set is shown in Fig. 5c (see Fig. A1 for all 14 replicates). The bivalent binding model fits the data well except for the minor deviation at the end of the association phase for the highest concentration (1 × 10^−6^ M) (Fig. 5c).

**Fig. 6:**
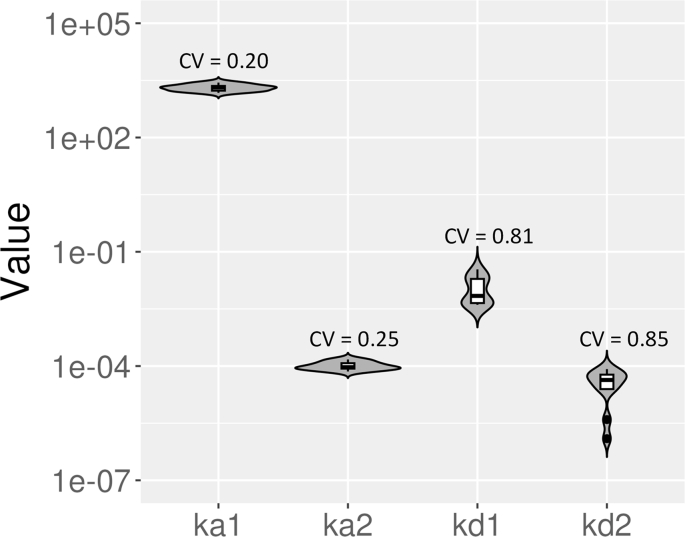
**Violin log-plots for estimated parameter of the bivalent analyte model for standard length of dissociation.** We illustrate the dispersion of the estimated parameters for the bivalent analyte model for standard length of dissociation using violin log-plots. From left to right, we show the violin log-plots for *k*_*a*1_, *k*_*a*2_, *k*_*d*1_, and *k*_*d*2_. In addition, we provide the computed coefficient of variation (CV) for each parameter. Note that the estimated parameters for data sets 9 and 10 are not included because the recovered dynamics are 1:1 Langmuir interactions. The estimated values for *k*_*d*2_ for datasets 1 and 3 are also excluded since log_10_(0) = −*∞*.

In Fig. 6, we show violin log-plots for the estimated kinetics parameters for data sets with standard length of dissociation for the bivalent analyte model (see Table A1 for full details). Based on Fig. 6, we found that the estimated values for *k*_*a*1_ and *k*_*a*2_ are consistent for most of the data sets after performing global fitting on 5 concentrations for each data set. Most of estimated *k*_*a*1_ values are of order of magnitude 3 and *k*_*a*2_ consistently estimated at about 10^−4^ *RU*^−1^*s*^−1^ (see Table A1). On the other hand, the order of magnitude for *k*_*d*1_ values are are less consistent and are estimated to between −3 and −2 (see Table A1). For *k*_*d*2_, the values for data sets 1 and 3 are estimated to be the lower bound 0 while their standard errors are 3.56 × 10^−6^ *s*^−1^ and 3.11 × 10^−6^ *s*^−1^, respectively (see Table A1). Furthermore, for each violin log-plot, we provide an associated coefficient of variation (CV), which can be computed by dividing the standard deviation for each parameter by the mean of each parameter across the data sets. The computed CV for *k*_*a*1_, *k*_*a*2_, *k*_*d*1_, and *k*_*d*2_ are 0.20, 0.25, 0.81, and 0.85, respectively. This result suggests that the estimated values for *k*_*a*1_ and *k*_*a*2_ are less disperse compared to *k*_*d*1_ and *k*_*d*2_. This led to our hypothesis that the dissociation rate constants might be practically non-identifiable with the current standard length of dissociation.

### 3.4 Log profile likelihood finds *k*_*d*2_ is a non-identifiable parameter

Parameter identifiability problems occur when multiple values of one or more parameters produce the same fitting error. Non-identifiable parameters may arise for many reasons, including when the number of parameters is too large or when there are too many unobservable states. We carried out parameter identifiability analysis to examine whether parameters can be uniquely estimated given the model and the data.

We note that there are two types of identifiability: structural, where the model itself is the source of non-identifiability [[23], [24], [25], [26], [27],^35^], and practical, where where the source of non-identifiability is the presence of noise or the lack of sufficient information in the data [24,26,35].

Structural identifiability issues occur when some model parameters are functions of the others, and may vary freely without changing the model output. Because our model is simple and parameters should be independent of one another, we assumed that our identifiability issue is practical. However, if there were structural identifiability issues, those would also be revealed with the profile likelihood method, therefore we can safely focus on practical identifiability.

To address practical identifiability we can either reduce noise or increase the information in the data. There is an inherent noise level in the SPR platform used, so reducing the noise level is not possible here. Instead, we tried to determine what additional data is needed in order to provide sufficient information for the algorithm to uniquely estimate parameters.

We hypothesized that one or both of the dissociation parameters might be practically non-identifiable with the standard length of dissociation. To investigate our hypothesis, we carried out parameter identifiability analysis using the log profile likelihood method as described in Section 2.4. This method has been applied previously to simulated synthetic data of a vector-borne disease model [24,25] and to a pharmacodynamics model [36]. First, we simulated synthetic data for the standard length of dissociation. The parameter values used to generate synthetic data are displayed in Table 3.

**Table 3:** **Parameter values for simulations.** Parameter values used for simulation to study parameter identifiability analysis. Using these values, we generated synthetic noisy data with standard (600 s) and extended (1780 s) length of dissociation.

Parameter	*k*_*a*1_	*k*_*d*1_	*k*_*a*2_	*k*_*d*2_	*R*max_1, *…*,5_	$t_{1,\ldots ,5}^{*}$
Value	2.00 × 10^3^	4.00 × 10^−3^	5.00 × 10^−5^	1.00 × 10^−5^	600	(283, 300, 282, 222, 145)

**Fig. 7:**
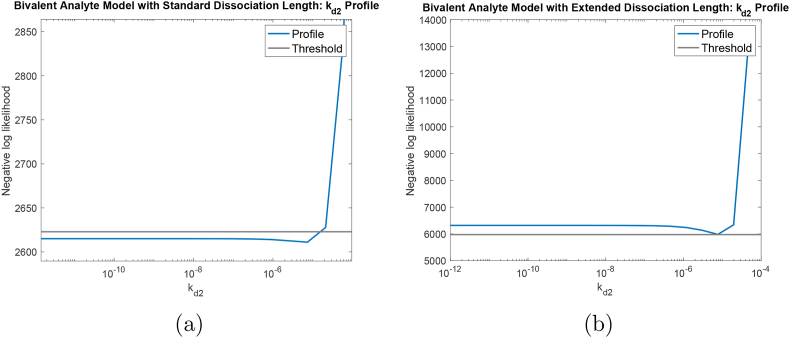
**Parameter identifiability analysis on synthetic noisy data.** Profile likelihood for *k*_*d*2_ with: (a) **standard** length of dissociation and (b) **extended** length of dissociation. For **standard** length of dissociation (a), the profile for *k*_*d*2_ resembles an *L*-shape with the left side stays below the threshold. This indicates that *k*_*d*2_ is not practically identifiable with the standard length of dissociation. For the **extended** length of dissociation (b), the left side of the profile for *k*_*d*2_ stays above the threshold indicating that *k*_*d*2_ is practically identifiable.

To simulate experimental data, we used the mathematical model in Section 2.2 and added normally distributed noise, $\epsilon ∼N\left(\mu ,\sigma^{2}\right)$ with *μ* = 0 and $\sigma =\sqrt{6}$. We note that all these values are chosen based on the results of parameter estimation on the experimental data with the standard length dissociation. In addition, *σ*^2^ = 6 corresponds to 2%–6% of the maximum response depends on the sensorgram. This satisfies the recommendation of having residual values being less than 10% of the maximum response of the fitted curve for a quality fit [30]. After generating synthetic noisy data, we analyzed the identifiability for kinetics parameters, *k*_*a*1_, *k*_*a*2_, *k*_*d*1_ and *k*_*d*2_. In Fig. 7a, we displayed the computed profile likelihood for *k*_*d*2_ with standard length of dissociation. In this figure, *k*_*d*2_ profile resembles the practically non-identifiable example being shown in Fig. 3a, *i.e.*, the profile is shallow on one side, particularly, on the left side in this case. This means, regardless of *k*_*d*2_ values on the left side, the algorithm is almost always able to achieve the global minimum error. This observation further explains our results for estimated values of *k*_*d*2_ in Table A1.

**Fig. A.2:**
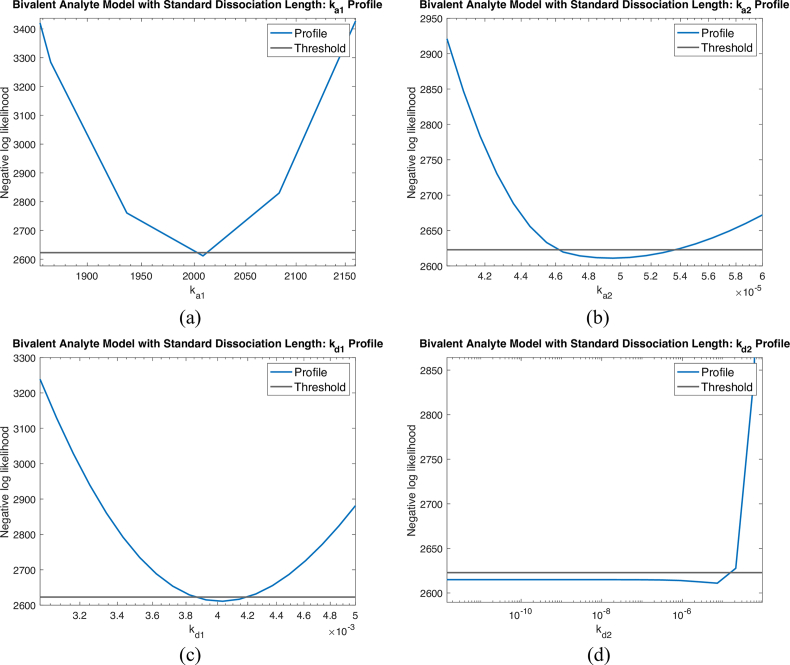
**Profile likelihood for kinetics parameters k_a1_, k_d1_, k_a2_, and k_d2_ on synthetic noisy data with standard length of dissociation.** Parameter identifiability analysis results on synthetic noisy data with standard length of dissociation. We use negative log profile likelihood as the method to perform parameter identifiability analysis on 4 kinetics parameters: (a) *k*_*a*1_, (b) *k*_*d*1_, (c) *k*_*a*2_, and (d) *k*_*d*2_.

On the other hand, the profiles for the remaining kinetics parameters, *k*_*a*1_, *k*_*a*2_, and *k*_*d*1_, manifest the bowl shape example in Fig. 3b (See Fig. A2). These profiles reinforce the observation that the estimated values for *k*_*a*1_, and *k*_*a*2_ are stable. While *k*_*d*1_ is somewhat unstable, it does meet the threshold for practical identifiabilty, and the highly variable *k*_*d*2_ is not identifiable.

**Fig. 8:**
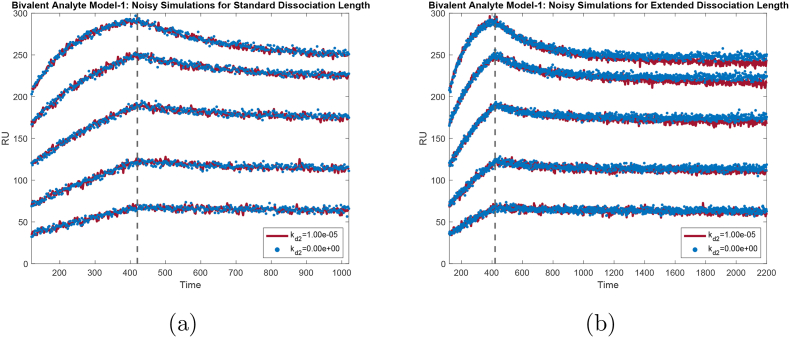
**Comparison of noisy simulated synthetic data.** Comparison plots of noisy simulations for: (a) **standard** dissociation length and (b) **extended** dissociation length. Each figure shows the noisy simulated solutions of the bivalent analyte binding model with *k*_*a*1_ = 2.00 × 10^3^ *M*^−1^*s*^−1^, *k*_*d*1_ = 4.00 × 10^−3^ *s*^−1^, *k*_*a*2_ = 5.00 × 10^−5^*RU*^−1^*s*^−1^, and two different values for *k*_*d*2_: **(red)** 1.00 × 10^−5^ *s*^−1^ and **(blue)** 0 *s*^−1^. For the **standard** length of dissociation (a), the noisy simulated solutions for both *k*_*d*2_ = 1.00 × 10^−5^ *s*^−1^ and *k*_*d*2_ = 0 *s*^−1^ are indistinguishable. On the other hand, for the **extended** dissociation length (b), the noisy simulated solution of the bivalent analyte binding model with *k*_*d*2_ = 1.00 × 10^−5^ *s*^−1^ can be distinguished from the same model solution with *k*_*d*2_ = 0 *s*^−1^.

To further reinforce our results, we simulated two sets of noisy synthetic data with the standard length of dissociation with *k*_*d*2_ = 10^−5^ *s*^−1^ and *k*_*d*2_ = 0 *s*^−1^. Note that all other parameters were kept the same as described in Table 3. In Fig. 8a, the fuzzy red curves represent the synthetic noisy data with *k*_*d*2_ = 10^−5^ *s*^−1^ for 5 concentrations while the scatter blue curves correspond to the synthetic noisy data with *k*_*d*2_ = 0 *s*^−1^. Even though the two values for *k*_*d*2_ are on different orders of magnitude, the dissociation phases are almost identical in the presence of noise in Fig. 8a. Based on these results, we concluded that *k*_*d*2_ is practically non-identifiable with the standard length of dissociation.

**Fig. D.1:**
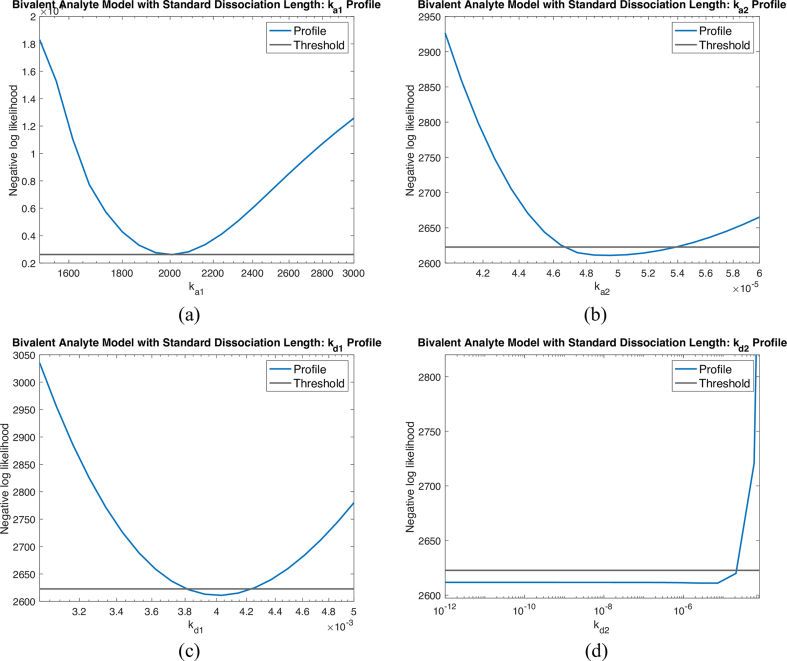
**Profile likelihood for kinetics parameters*****k***_***a*****1**_**,*****k***_***d*****1**_**,*****k***_***a*****2**_**, and*****k***_***d*****2**_**on synthetic noisy regenerative titration data with standard length of dissociation.** Parameter identifiability analysis results on synthetic noisy data with standard length of dissociation. We use negative log profile likelihood as the method to perform parameter identifiability analysis on 4 kinetics parameters (a) *k*_*a*1_, (b) *k*_*d*1_, (c) *k*_*a*2_, and (d) *k*_*d*2_.

**Fig. D.2:**
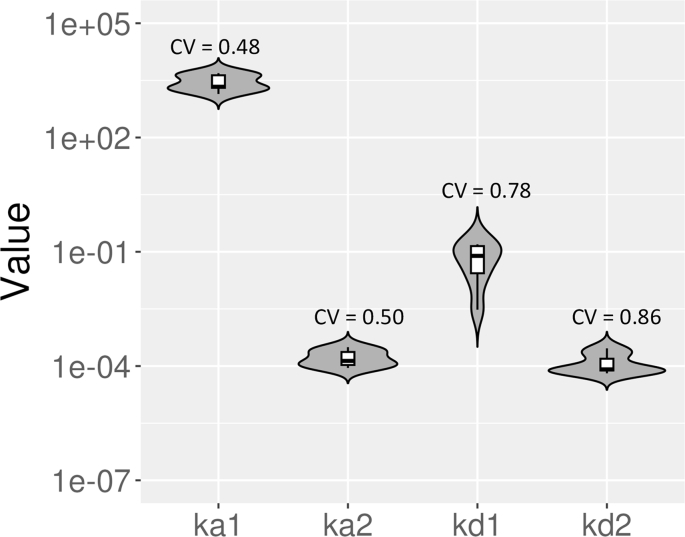
**Violin log-plots for estimated parameters of the bivalent analyte model for regenerative titration with standard length of dissociation.** We illustrate the dispersion of the estimated parameters for the bivalent analyte model for regenerative titration with standard length of dissociation using violin log-plots. From left to right, we show the violin log-plots for *k*_*a*1_, *k*_*a*2_, *k*_*d*1_, and *k*_*d*2_. In addition, we provide the computed coefficient of variation (CV) for each parameter.

We further used regenerative titration to verify that the source of the non-identifiability issue with *k*_*d*2_ is not over-parameterization, *i.e.*, fitting additional parameters due to non-regenerative titration. We performed parameter identifiability analysis on a simulated data set with regenerative titration and obtained parameter estimates on experimental data from regenerative titrations with the standard length of dissociation. In Fig. D1, we show that even without the additional parameters for initial time adjustment, *k*_*d*2_ remained practically non-identifiable for the simulated data with the standard length of dissociation. Moreover, we found that for experimental data from regenerative titrations, the estimated values for *k*_*d*1_ and *k*_*d*2_ vary on multiple orders of magnitude (See Table D1). Fig. D2 shows that the estimates for dissociation rate constants *k*_*d*1_ and *k*_*d*2_ were more dispersed and were associated with higher CV values when compared to the estimates for association rate constants *k*_*a*1_ and *k*_*a*2_, with *k*_*d*2_ having the highest CV number. These results suggest that, with standard length dissociation, *k*_*d*2_ is practically non-identifiable even under regenerative conditions.

**Fig. D.3:**
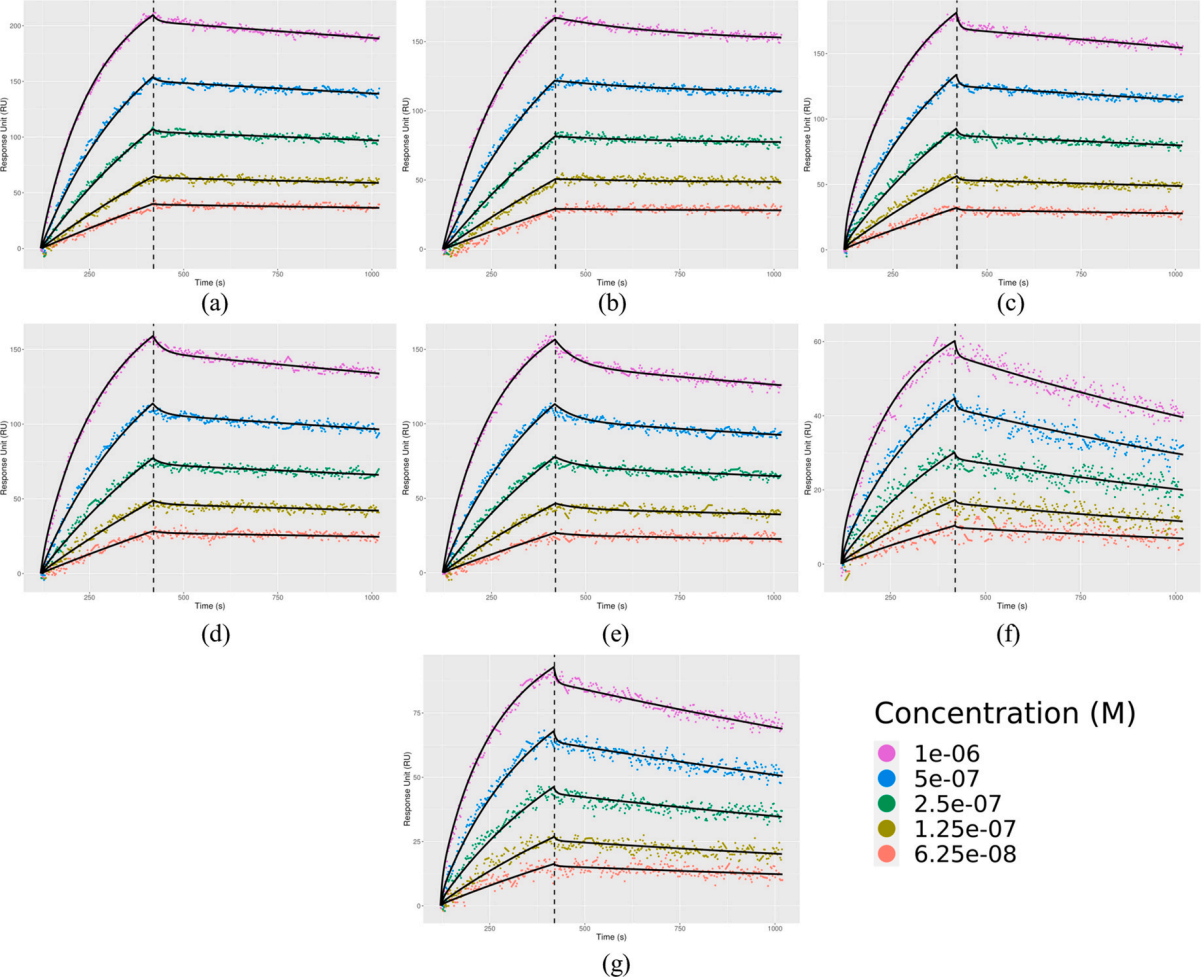
**Bivalent analyte model fitting results for regenerative titration data sets with the standard length of dissociation.** Model fitting results using the bivalent analyte model for: (a)–(g) regenerative titration data sets 1–7 with standard length of dissociation.

During non-regenerative titrations, the initial curvature of the association phase is affected by the analyte remaining bound prior to the start of the association phase. As a result, we also aimed to use regenerative titration data to verify the validity of the association rate estimates from non-regenerative titration data. As shown in Fig. D2, D3 and Table D1, we found that the difference in curvature did not affect the accuracy of *k*_*a*1_ and *k*_*a*2_ estimates.

### 3.5 Simulation guides identifying optimal experimental conditions

As suggested in previously published literature [24,26,35], parameter identifiability may be improved by collecting additional data, in order to provide sufficient information for the algorithm to uniquely estimate parameters. While it is not possible to collect more data by increasing the sampling frequency due to the limitation of our equipment, collecting additional data by increasing the length of dissociation phase is achievable. It is possible to collect experimental data multiple times to incrementally include more temporal points each iteration, but this is certainly not optimal in terms of time and resource efficiency. Instead, we use synthetic data from simulation to find the optimal experimental setup. This approach greatly reduce the need to repeatedly collect experimental data.

We examined parameter identifiability on the same synthetic data, but with extended length of dissociation phase. Here, we chose the length of dissociation to be 1780 s. The profile likelihood for *k*_*d*2_ is shown in Fig. 7b. Identifiability analysis showed that with the extended length of dissociation, *k*_*d*2_ is practically identifiable as both sides of *k*_*d*2_'s profile are non-flat according its corresponding computed threshold. We again further reinforced our conclusion by simulating synthetic noisy data again for two different *k*_*d*2_ values, *k*_*d*2_ = 10^−5^ *s*^−1^ (fuzzy red curves) and *k*_*d*2_ = 0 *s*^−1^ (scatter blue curves), with extended length of dissociation. In Fig. 8b, we displayed the comparison for such simulations. We found that the distinction between the two simulated noisy synthetic data are much more noticeable compared to the case with standard length of dissociation. For other kinetics parameters, their profiles preserve their practical identifiability as shown in Fig. B2.

In conclusion, all kinetics parameters, including *k*_*d*2_, are practically identifiable in the simulated data with extended length of dissociation.

### 3.6 Kinetics parameters can be reliably estimated with extended length of dissociation

**Fig. 9:**
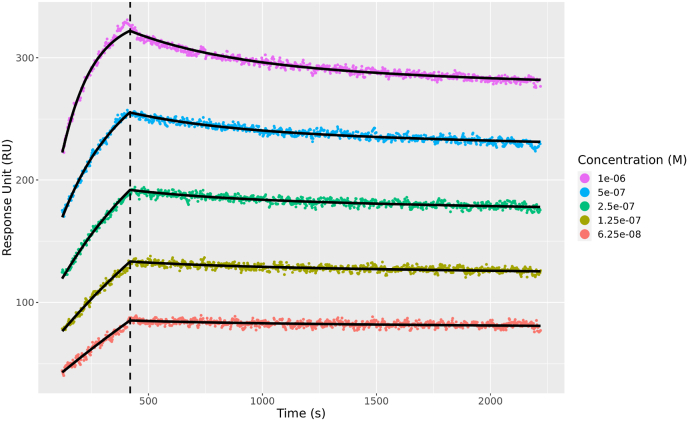
**A representative bivalent analyte model fitting result with extended length of dissociation.** Bivalent analyte model fitted sensorgrams of CH31 mAb binding to CH505 T/F gp120 are shown. The **color scatter** curves are the data for five different concentrations of the same interaction with the concentration values are shown in the legend. The **black solid** curves are the fitting results using the model. The vertical **black dashed** line separates the association and dissociation phases.

**Fig B.1:**
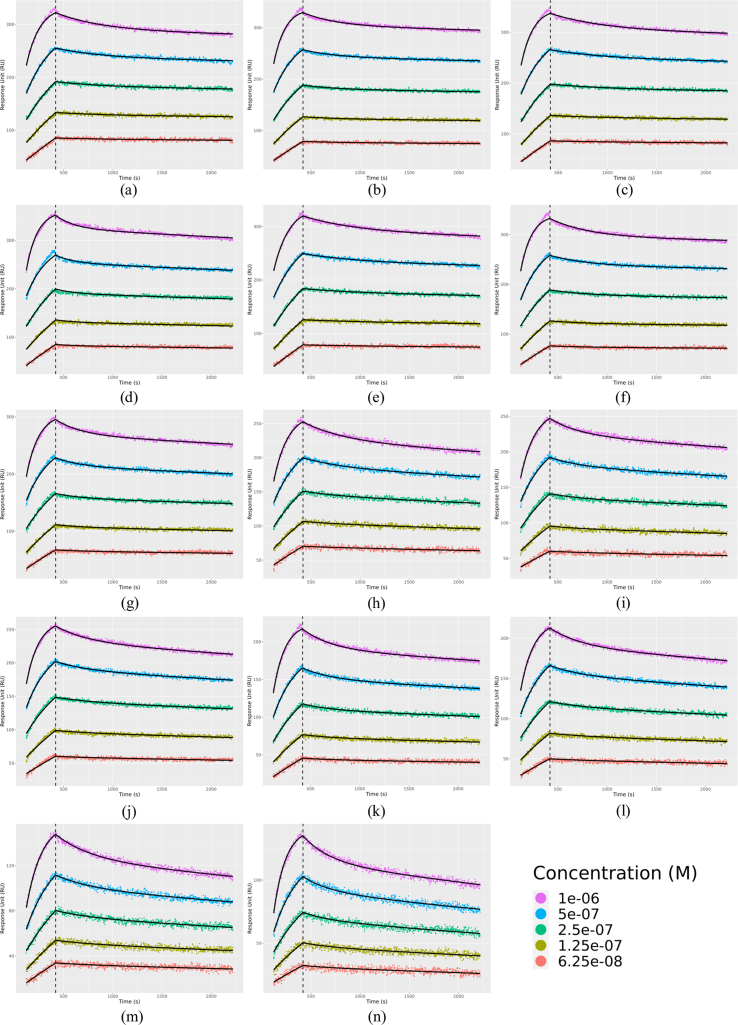
**Bivalent analyte model fitting results for the extended length of dissociation.** Model fitting results using the bivalent analyte model for: (a)–(n) data sets 1–14 with extended length of dissociation.

**Fig. B.2:**
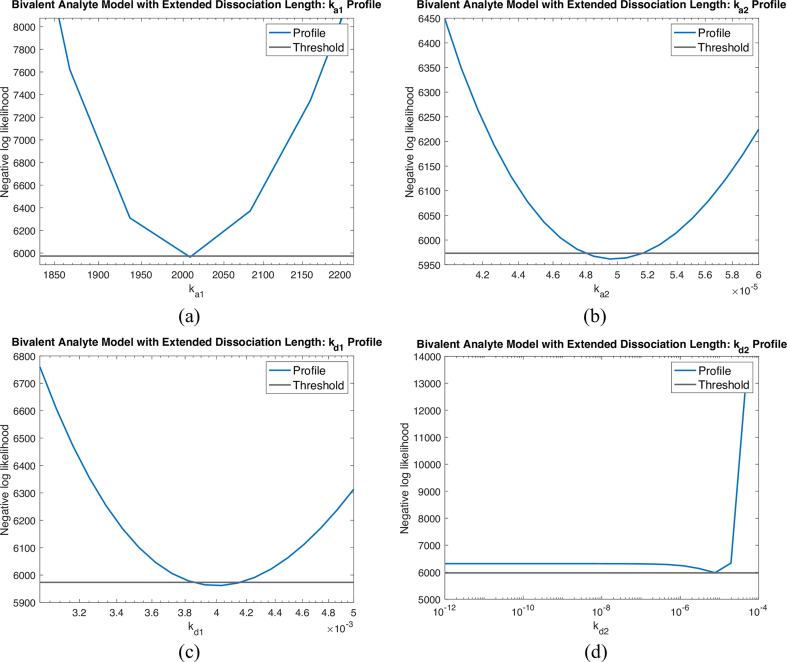
**Profile likelihood for kinetics parameters*****k***_***a*****1**_**,*****k***_***d*****1**_**,*****k***_***a*****2**_**, and*****k***_***d*****2**_**on synthetic noisy data with extended length of dissociation.** Parameter identifiability analysis results on synthetic noisy data with extended length of dissociation. We use negative log profile likelihood as the method to perform parameter identifiability analysis on 4 kinetics parameters (a) *k*_*a*1_, (b) *k*_*d*1_, (c) *k*_*a*2_, and (d) *k*_*d*2_.

As demonstrated in Section 3, 3.4.5, all kinetic parameters for the bivalent analyte model are practically identifiable with the extended length of dissociation using noisy synthetic data. In this section, we discussed our parameter estimation results on experimental data with extended length of dissociation. In Fig. 9, we show the results for the first data set as a representative example (see Fig. B1 for all 14 replicates). Similar to the results in Fig. 5c (see Fig. A1 for all 14 replicates) with the standard length of dissociation, the bivalent analyte model is able to describe the interaction between CH31 mAb and CH505 T/F gp120 (see Fig. B1 for all 14 replicates).

**Fig. 10:**
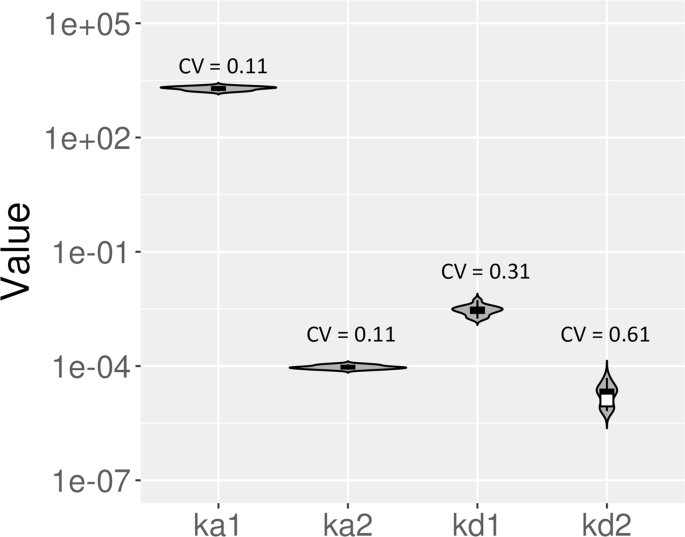
**Violin log-plots for estimated parameter of the bivalent analyte model for extended length of dissociation.** The log-plots illustrate the dispersion of the estimated parameters for the bivalent analyte model for extended length of dissociation. From left to right, the violin log-plots for *k*_*a*1_, *k*_*a*2_, *k*_*d*1_, and *k*_*d*2_ are shown. The computed coefficient of variation (CV) for each parameter is labeled above the corresponding log-plot. The estimated parameters for all data sets are included in the distributions.

As shown in Fig. 10, the values for *k*_*d*2_ of the bivalent analyte model with the extended length of dissociation are more consistently estimated compared to the results with the standard length of dissociation Fig. 6 (also, see Table A1 And Table B1.). In addition to the improvement in *k*_*d*2_ identifiability, the extended dissociation length improves the consistency of estimates of *k*_*d*1_ as shown in Fig. 10. Furthermore, the computed CV for *k*_*d*1_ and *k*_*d*2_ decrease from 0.81 to 0.31 and 0.85 to 0.61, respectively.

Furthermore, by extending the dissociation length, *k*_*d*2_ can be reliably estimated to be about 10^−5^ as shown in Table B1. We also see the improvement in consistency in estimation of other kinetics parameters. For example, in Table A1, *k*_*d*1_ values are estimated to be between 10^−3^ *s*^−1^ and $>10^{-2}\;s^{-1}$ for data sets with the standard length of dissociation. In contrast, for the extended length of dissociation, *k*_*d*1_ are consistently estimated to be approximately 3 × 10^−3^ *s*^−1^ (see Table B1). Note that the replicates included here correspond to a wide range of ligand surface density (1250–2700 RU, see Table B2) and top binding response (120–360 RU), indicating that varying ligand surface density in this range does not significantly change the bivalent binding behavior.

**Fig. D.4:**
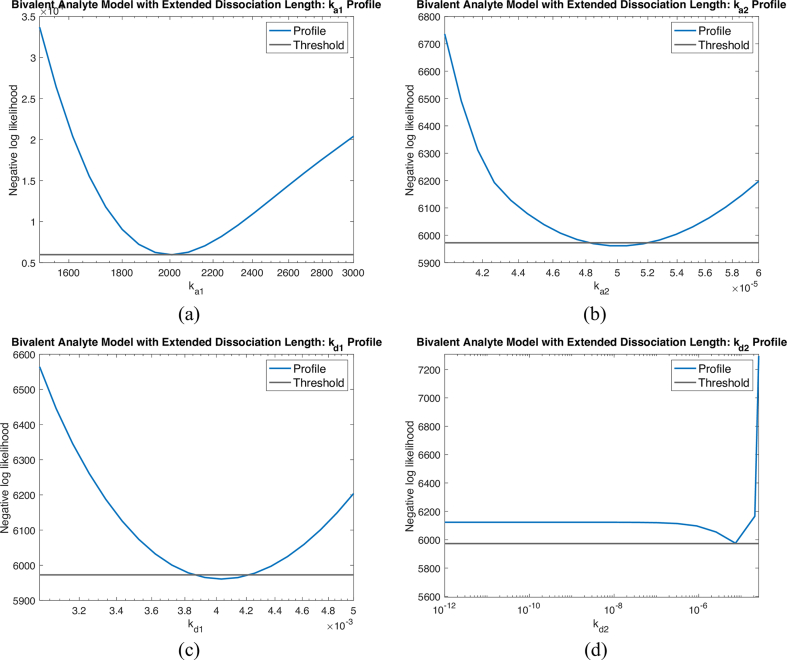
**Profile likelihood for kinetics parameters*****k***_***a*****1**_**,*****k***_***d*****1**_**,*****k***_***a*****2**_**, and*****k***_***d*****2**_**on synthetic noisy regenerative titration data with extended length of dissociation.** Parameter identifiability analysis results on synthetic noisy data with extended length of dissociation. We use negative log profile likelihood as the method to perform parameter identifiability analysis on 4 kinetics parameters (a) *k*_*a*1_, (b) *k*_*d*1_, (c) *k*_*a*2_, and (d) *k*_*d*2_.

**Fig. D.5:**
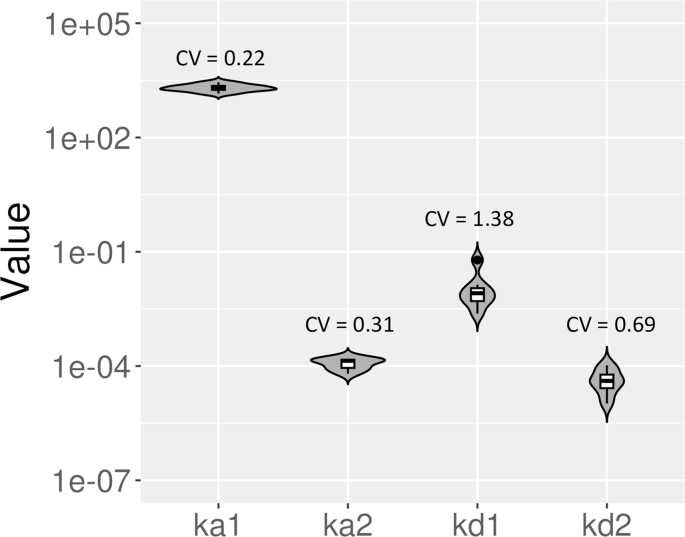
**Violin log-plots for estimated parameters of the bivalent analyte model for regenerative titration with extended length of dissociation.** We illustrate the dispersion of the estimated parameters for the bivalent analyte model for regenerative titration with extended length of dissociation using violin log-plots. From left to right, we show the violin log-plots for *k*_*a*1_, *k*_*a*2_, *k*_*d*1_, and *k*_*d*2_. In addition, we provide the computed coefficient of variation (CV) for each parameter.

**Fig. D.6:**
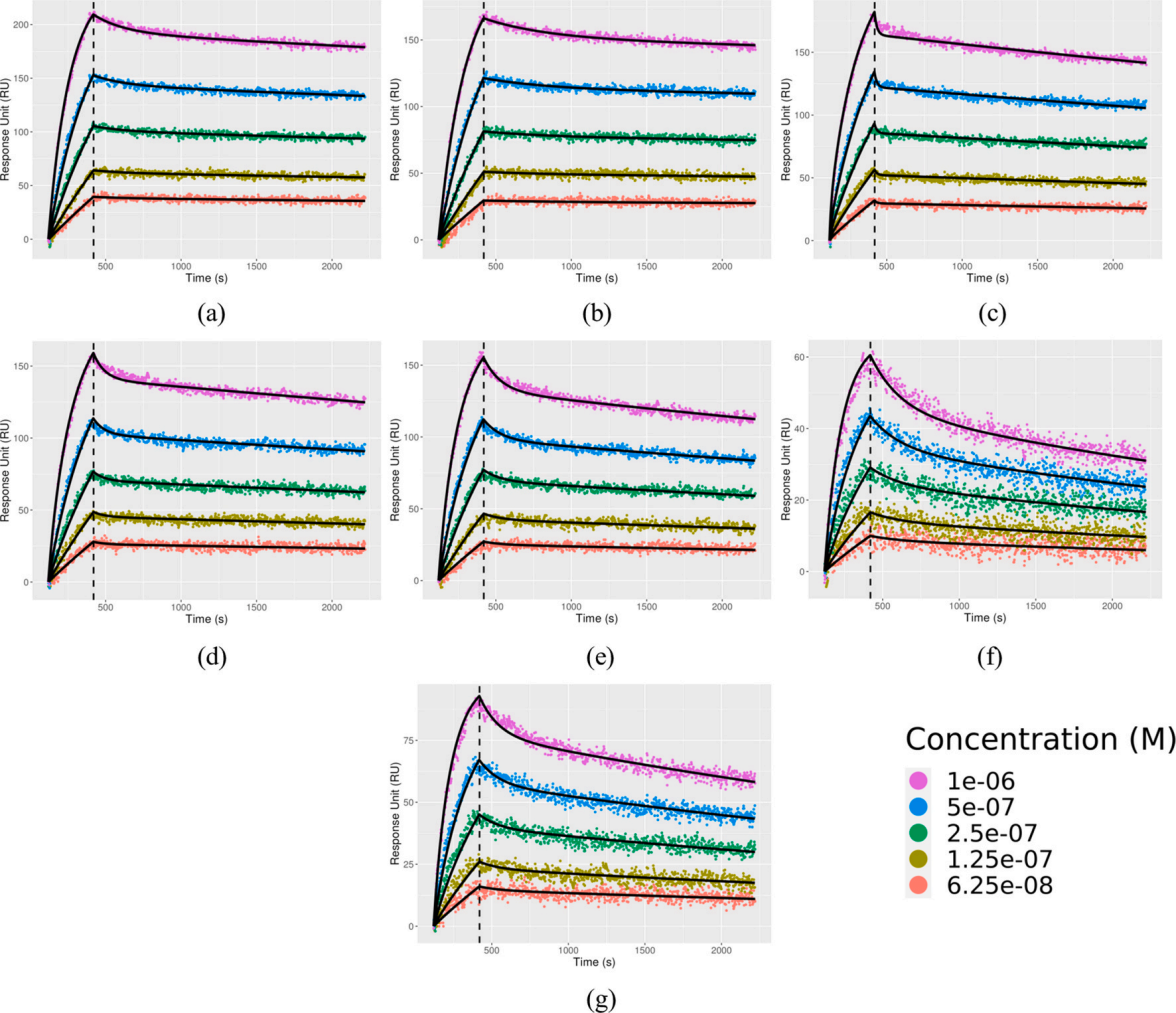
**Bivalent analyte model fitting results for regenerative titration data sets with the extended length of dissociation.** Model fitting results using the bivalent analyte model for: (a)–(g) regenerative titration data sets 1–7 with extended length of dissociation.

The benefit of using the extended dissociation length was also verified using regenerative titrations. Here, similar to non-regenerative titrations, *k*_*d*2_ becomes identifiable with extended length dissociation (Fig. D4). The orders of magnitude for *k*_*d*1_ and *k*_*d*2_ also became more consistent with the extended length of dissociation (Figs. D5, D6 and Table D2) as compared to the standard length of dissociation (Figs. D2, D3 and Table D1), while the estimates for *k*_*a*1_ and *k*_*a*2_ remained largely unaffected.

We further compared the parameter estimates from our fitting results to those of the bivalent analyte model fitting using Biacore BIAevlauation software. Due to the difference in how Carterra and Biacore handle non-regenerative titration data collection and analysis, we were not able to directly compare parameters from non-regenerative titration data. Instead, we analyzed the regenerative titration data (Figs. D3, D6) in BIAevlauation software.

**Fig. E.1:**
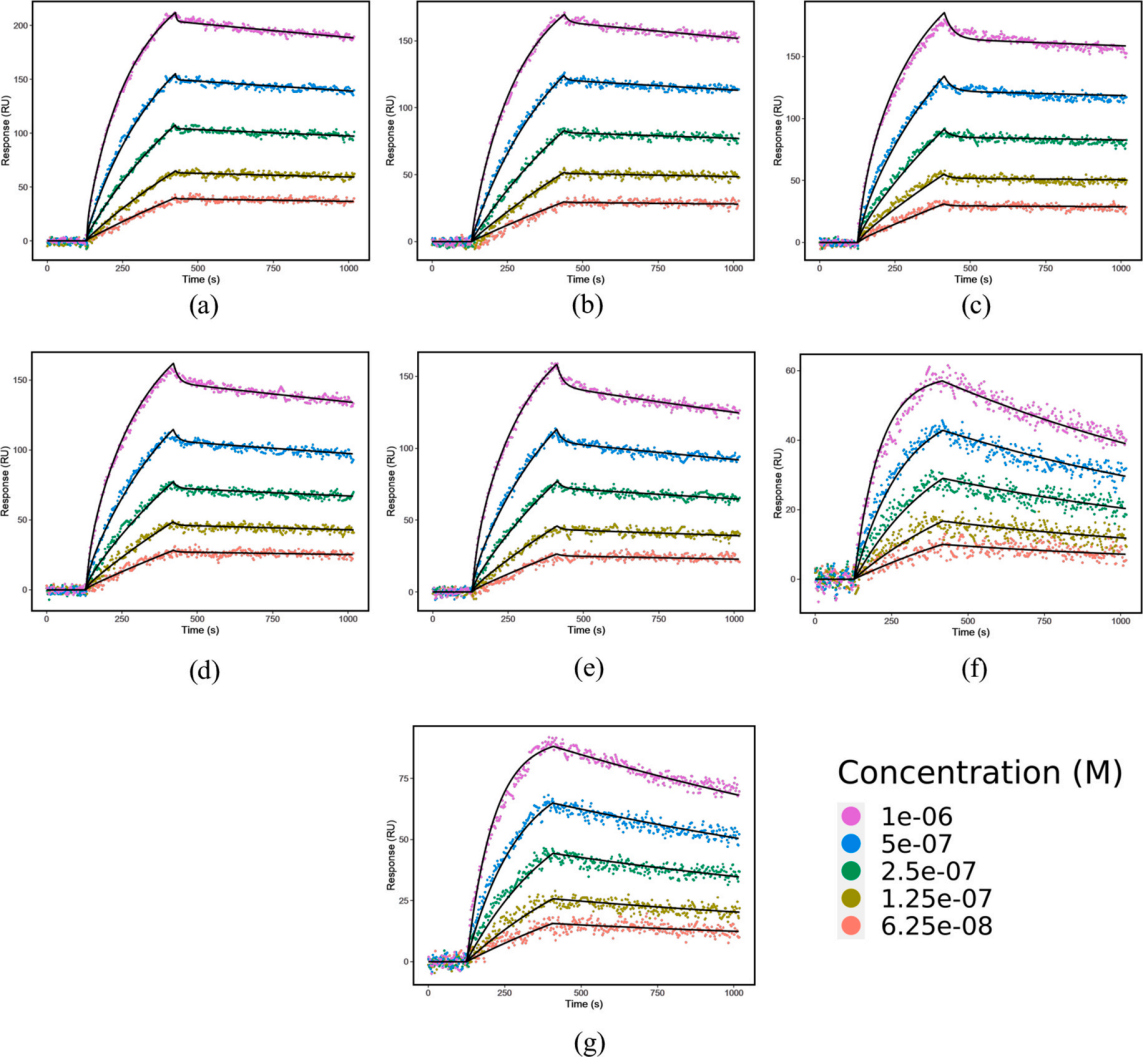
**Bivalent analyte model fitting results for regenerative titration data sets with the standard length of dissociation using BIAevaluation software.** Model fitting results using the bivalent analyte model for: (a)–(g) regenerative titration data sets 1–7 with standard length of dissociation using BIAevaluation software.

**Fig. E.2:**
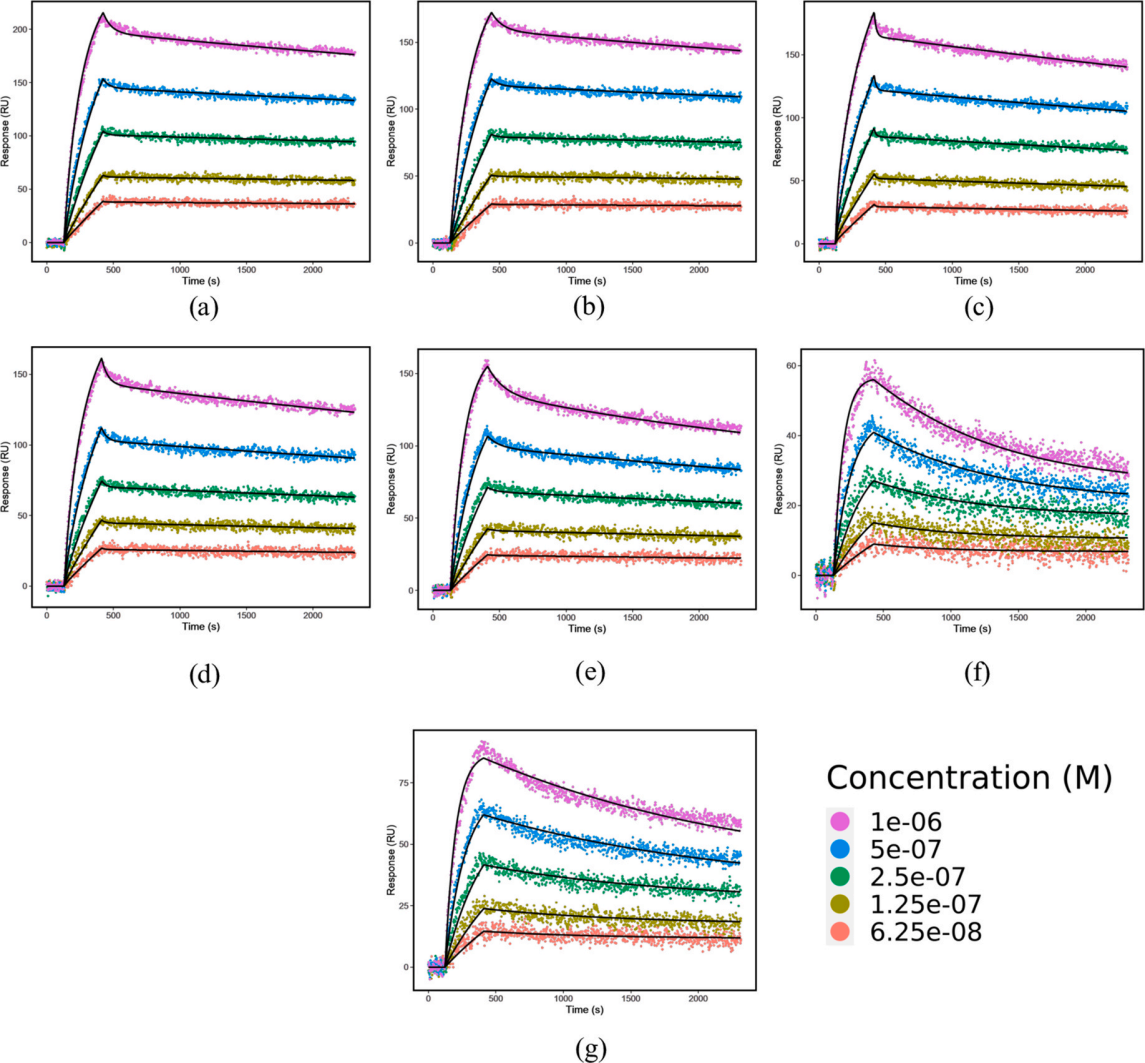
**Bivalent analyte model fitting results for regenerative titration data sets with the extended length of dissociation using BIAevaluation software.** Model fitting results using the bivalent analyte model model for: (a)–(g) regenerative titration data sets 1–7 with extended length of dissociation using BIAevaluation software.

As shown in Figs. E1, E2 and Table E1, E2, analysis in BIAevlauation using both standard length dissociation and extended length dissociation produced *k*_*a*1_ and *k*_*a*2_ estimates that were comparable to those from our bivalent analyte model (Table. A.1, Table. B.1, Table. D.1, Table. D.2). For high response sensorgrams (Fig. E1a-e), estimates for *k*_*d*2_ using standard length dissociation through BIAevlauation were also comparable to standard length analysis using our model (Table. A.1, Table. D.1). When analyzing extended length dissociation, BIAevlauation slightly overestimated the slope of decay for *k*_*d*2_, but the range of *k*_*d*2_ was in general agreeing with the range of *k*_*d*2_ for data analyzed using our model (Table. B.1, Table. D.2).

## 4 Discussion

Biophysical determinations of antibody-antigen interactions directly inform selection of mAbs for immunoprophylaxis trials and novel immunogen design. For SPR data of antigen binding to immobilized mAbs, the 1:1 Langmuir binding model is appropriate [[8], [9], [10], [11]]. However, when using mAbs as analyte binding to antigens, a bivalent analyte binding model is required to better describe the kinetics data unless the antigen is immobilized to an optimal density to eliminate avidity effects.

Values for *k*_*a*1_ and *k*_*d*1_ describe the innate ability of each arm of the antibody to interact with the antigen, while *k*_*a*2_ and *k*_*d*2_ are incorporated in the bivalent analyte model to describe data accounting for the avidity of the antibody binding to immobilized antigen. Therefore, *k*_*a*2_ and *k*_*d*2_ reflect the efficiency of second-arm binding of the antibody. The extent of this avidity effect is influenced by multiple factors, including the surface density of ligands in the vicinity of monovalent bound antibody-ligand complex that can be accessed by the second arm of the antibody and the binding mode of the antibody [37,38]. Therefore, same or similar ligand densities should be used when comparing *k*_*a*2_ and *k*_*d*2_ for different antibodies for their cross-arm binding efficiency. Knowledge on these parameters from antibody interaction analysis with live cell surface antigens will be valuable in interpreting target antigen occupancy of therapeutic antibodies [39,40] and to design for enhanced cross-arm binding efficiency.

**Fig. F.1:**
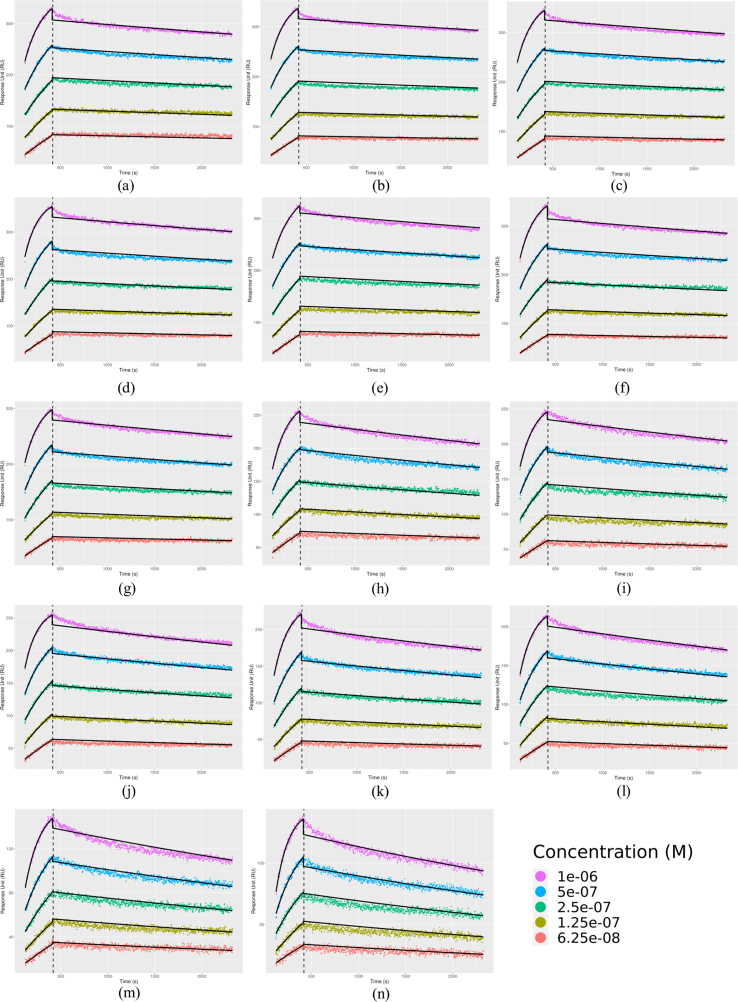
**1:1 Langmuir model fitting results for the extended length of dissociation using Kinetics software.** Model fitting results using the 1:1 Langmuir model for: (a)–(n) data sets 1–14 with extended length of dissociation using Kinetics (Carterra platform analysis software).

Existing models and implementations [14,[41], [42], [43], [44]] cannot be easily applied to high-throughput, non-regenerative titration data, and they do not address local minima or parameter identifiability. Currently, the commercial software of the Carterra platform (Kinetics) is also not capable of fitting for bivalent analyte model. The 1:1 Langmuir model fitting results with extended length of dissociation using Carterra software (Fig. F1) failed to capture the underlying two-step process, with the fitted curves in the dissociation phase appearing relatively flat. This highlights the need to develop a rigorous model for bivalent analyte binding.

In this work, we have introduced a robust parameter estimation pipeline for a bivalent analyte model that can be applied to high-throughput data and that directly addresses the problems of local minima and parameter identifiability. We further used our identifiability analysis to optimize the experimental design. Because the parameter in question was the second dissociation rate constant, we simulated experiments with extended length of the dissociation phase and proposed an optimal duration of observation. We then collected data under the proposed design and succeeded in identifying all of the model parameters. Simulation, together with identifiability analysis can save time, materials and funds, by providing information about experimental variables such as length of dissociation data collection.

We used a common local optimization algorithm, the Levenberg–Marquardt algorithm [34]. Our results from the grid search (see Table 2) show that with the “wrong” (yet physically reasonable) initial guess, it is possible to report results at a local minimum. Different kinetic interactions will have different optimal starting values. We therefore used a grid of 81 initial values. This process is very computationally expensive and inefficient: The full analysis took approximately 4 h on server running R.1.4.17 [45] using parallel computing with 14 cores and 16 GB RAM. For future work, we will consider methods to better select initial guesses or implement a global optimization algorithm [46,47].

The parameter estimation and grid search are currently implemented in R [45] and are therefore open source. The profile likelihood analysis is implemented in MATLAB. We plan to include the profile likelihood in R to include in our package that is under development.

## 5 Conclusion

In this work, we introduce a pipeline for analysis of bivalent analyte binding kinetics that is effective for high-throughput, non-regenerative experimental designs and offers reliable parameter estimation through parameter initialization grid search and parameter identifiability analysis using profile likelihood. We were able to combine simulation and identifiability analysis to further determined the optimal length of dissociation so all kinetics parameters can be reliably estimated, saving time and reagents. These methodologies offer robust determination of the kinetics parameters for high-throughput bivalent analyte SPR experiments.

## Funding

This study was supported by a grant (INV-0008612) for the Antibody Dynamics platform of the Global Health Discovery Collaboratory (GHDC) from the 10.13039/100000865Bill and Melinda Gates Foundation (10.13039/100000865BMGF) to GDT and partly supported by a grant for the Antibody Dynamics platform of the Global Health – Vaccine Accelerator Platforms (GH-VAP) from the 10.13039/100000865BMGF to GDT (OPP12109388), an 10.13039/100000060NIAID education research program (R25AI140495) and the 10.13039/100006967Duke University Center for AIDS Research (CFAR)(5P30 AI064518). KN was supported by the 10.13039/100000001National Science Foundation Graduate Research Fellowship under Grant No. DGE-2137100. Any opinion, findings, and conclusions or recommendations expressed in this material are those of the authors and do not necessarily reflect the views of the funding sponsors.

## Author statement

Kyle Nguyen: Conceptualization, Formal analysis, Methodology, Software, Visualization, Writing – original draft, Writing – review & editing. Kan Li: Conceptualization, Data curation, Formal analysis, Methodology, Visualization, Writing – review & editing. Kevin Flores: Methodology, Writing – review & editing. Georgia D. Tomaras: Conceptualization, Funding acquisition, Project administration, Supervision, Writing – review & editing. S. Moses Dennison: Conceptualization, Investigation, Supervision, Writing – review & editing. Janice M. McCarthy: Conceptualization, Investigation, Methodology, Project administration, Software, Supervision, Writing – original draft, Writing – review & editing.

## Declaration of competing interest

The authors declare that they have no known competing financial interests or personal relationships that could have appeared to influence the work reported in this paper.

https://www.elsevier.com/declaration-of-competing-interests.

## Figures and Tables

**Table. A.1:** **Estimated kinetics parameters for bivalent analyte model for data sets with standard length of dissociation.** Estimated and standard error (SE) values for kinetics parameters, *k*_*a*1_, *k*_*a*2_, *k*_*d*1_, and *k*_*d*2_, for all 14 data sets.

	Bivalent Analyte Model							
	*k*_*a*1_(*M*^−1^*s*^−1^)		*k*_*a*2_(*RU*^−1^*s*^−1^)		*k*_*d*1_(*s*^−1^)		*k*_*d*2_(*s*^−1^)	
Data set	Estimated	SE	Estimated	SE	Estimated	SE	Estimated	SE
1	1.71E+03	3.62E+01	7.50E-05	4.14E-06	4.05E-03	2.73E-04	0.00	3.56E-06
2	2.22E+03	4.53E+01	1.08E-04	4.26E-06	1.76E-02	1.07E-03	2.68E-05	1.26E-06
3	1.88E+03	3.43E+01	8.97E-05	4.69E-06	4.65E-03	3.11E-04	0.00	3.11E-06
4	2.77E+03	8.11E+01	8.24E-05	3.40E-06	2.42E-02	1.61E-03	3.84E-05	1.41E-06
5	1.49E+03	3.19E+01	8.69E-05	5.59E-06	4.09E-03	3.29E-04	3.93E-06	3.39E-06
6	2.07E+03	3.55E+01	7.20E-05	3.21E-06	4.64E-03	2.54E-04	1.24E-06	3.47E-06
7	2.72E+03	9.99E+01	9.35E-05	4.03E-06	3.46E-02	2.57E-03	4.83E-05	1.21E-06
8	1.66E+03	4.18E+01	9.35E-05	5.64E-06	5.13E-03	3.68E-04	2.42E-05	3.43E-06
9	1.28E+04	4.58E+03	1.27E-04	8.74E-06	4.37E-01	1.72E-01	6.36E-05	1.20E-06
10	5.10E+03	6.57E+02	1.83E-04	1.12E-05	1.34E-01	2.45E-02	6.45E-05	9.91E-07
11	2.39E+03	6.21E+01	1.19E-04	5.13E-06	2.12E-02	1.48E-03	6.68E-05	1.79E-06
12	2.07E+03	5.20E+01	1.49E-04	7.49E-06	1.89E-02	1.48E-03	6.13E-05	1.67E-06
13	1.63E+03	4.01E+01	1.20E-04	8.16E-06	4.13E-03	3.15E-04	5.42E-05	5.17E-06
14	2.11E+03	5.08E+01	1.48E-04	8.01E-06	9.40E-03	6.77E-04	8.34E-05	3.96E-06

**Table. B.1:** **Estimatedkinetics parameters for bivalent analyte model for data sets with extended length of dissociation.** Estimated and standard error (SE) values for kinetics parameters, *k*_*a*1_, *k*_*a*2_, *k*_*d*1_, and *k*_*d*2_, for all 14 data sets.

	Bivalent Analyte Model							
	*k*_*a*1_(*M*^−1^*s*^−1^)		*k*_*a*2_(*RU*^−1^*s*^−1^)		*k*_*d*1_(*s*^−1^)		*k*_*d*2_(*s*^−1^)	
Data set	Estimated	SE	Estimated	SE	Estimated	SE	Estimated	SE
1	1.83E+03	3.48E+01	7.95E-05	2.08E-06	2.09E-03	5.71E-05	7.72E-06	5.03E-07
2	2.16E+03	3.24E+01	1.12E-04	2.59E-06	2.99E-03	8.44E-05	8.45E-06	3.75E-07
3	2.03E+03	3.23E+01	9.03E-05	2.16E-06	1.76E-03	4.87E-05	6.54E-06	4.93E-07
4	2.27E+03	3.96E+01	8.95E-05	2.54E-06	5.38E-03	1.69E-04	1.75E-05	3.36E-07
5	1.61E+03	3.09E+01	8.24E-05	2.33E-06	1.83E-03	5.70E-05	9.60E-06	5.11E-07
6	2.23E+03	3.41E+01	8.19E-05	1.77E-06	2.52E-03	6.00E-05	7.39E-06	4.84E-07
7	2.02E+03	3.68E+01	9.09E-05	2.42E-06	3.86E-03	1.11E-04	1.79E-05	4.18E-07
8	1.75E+03	3.99E+01	8.53E-05	2.61E-06	2.46E-03	7.00E-05	2.26E-05	5.36E-07
9	1.72E+03	4.35E+01	9.29E-05	3.22E-06	3.30E-03	1.13E-04	2.34E-05	5.37E-07
10	1.98E+03	3.46E+01	1.04E-04	2.60E-06	2.99E-03	7.90E-05	2.33E-05	4.30E-07
11	2.12E+03	3.55E+01	9.56E-05	2.25E-06	3.48E-03	8.62E-05	2.43E-05	5.37E-07
12	1.91E+03	3.62E+01	1.04E-04	2.75E-06	3.15E-03	8.39E-05	2.82E-05	4.94E-07
13	1.62E+03	3.95E+01	9.45E-05	2.97E-06	2.83E-03	7.88E-05	3.81E-05	7.80E-07
14	2.05E+03	4.61E+01	1.11E-04	3.37E-06	3.80E-03	1.07E-04	4.90E-05	7.62E-07

**Table. B.2:** **Printingdensity for all 14 data sets.**

Data set	Ligand density (RU)
1	2646
2	1899
3	2392
4	2516
5	2273
6	1972
7	2004
8	2675
9	2122
10	1180
11	1528
12	1234
13	1521
14	1282

**Figure:**
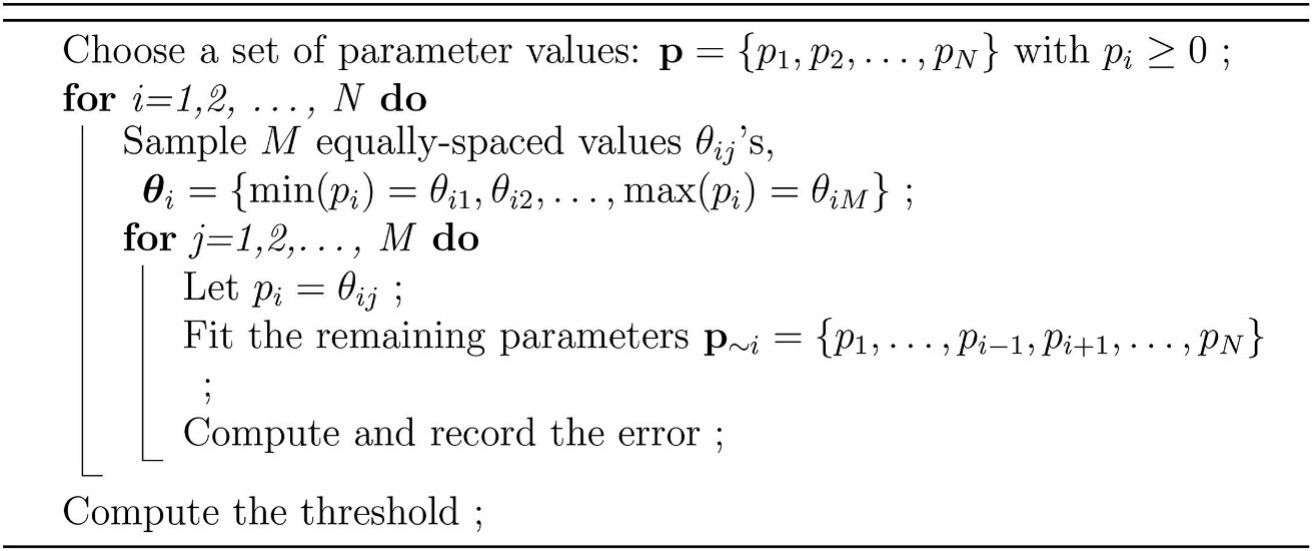


**Table. D.1:** **Estimated kineticsparametersfor bivalent analyte model for regenerative titration data sets with standard length of dissociation.** Estimated and standard error (SE) values for kinetics parameters, *k*_*a*1_, *k*_*a*2_, *k*_*d*1_, and *k*_*d*2_, for all 7 data sets.

	Bivalent Analyte Model							
	*k*_*a*1_(*M*^−1^*s*^−1^)		*k*_*a*2_(*RU*^−1^*s*^−1^)		*k*_*d*1_(*s*^−1^)		*k*_*d*2_(*s*^−1^)	
Data set	Estimated	SE	Estimated	SE	Estimated	SE	Estimated	SE
1	2.15E+03	6.76E+01	2.67E-04	2.62E-05	7.81E-02	1.12E-02	6.45E-05	1.92E-06
2	1.39E+03	1.89E+01	1.36E-04	2.34E-05	3.01E-03	6.78E-04	0.00	1.35E-05
3	3.97E+03	2.21E+02	9.25E-05	4.84E-06	1.32E-01	1.26E-02	7.53E-05	2.20E-06
4	2.07E+03	4.91E+01	1.23E-04	7.52E-06	4.11E-02	3.51E-03	8.25E-05	2.81E-06
5	1.96E+03	2.95E+01	8.89E-05	4.26E-06	1.81E-02	1.15E-03	8.12E-05	4.13E-06
6	4.66E+03	6.71E+02	3.14E-04	3.89E-05	1.57E-01	3.95E-02	2.92E-04	5.84E-06
7	4.91E+03	4.77E+02	2.05E-04	1.72E-05	1.51E-01	2.51E-02	1.93E-04	3.83E-06

**Table. D.2:** **Estimated kinetics parameters for bivalent analyte model for regenerative titration data sets with extended length of dissociation.** Estimated and standard error (SE) values for kinetics parameters, *k*_*a*1_, *k*_*a*2_, *k*_*d*1_, and *k*_*d*2_, for all 7 data sets.

	Bivalent Analyte Model							
	*k*_*a*1_(*M*^−1^*s*^−1^)		*k*_*a*2_(*RU*^−1^*s*^−1^)		*k*_*d*1_(*s*^−1^)		*k*_*d*2_(*s*^−1^)	
Data set	Estimated	SE	Estimated	SE	Estimated	SE	Estimated	SE
1	1.79E+03	1.42E+01	1.50E-04	4.23E-06	4.95E-03	2.50E-04	2.04E-05	6.09E-07
2	1.43E+03	1.46E+01	1.56E-04	6.23E-06	2.39E-03	1.65E-04	1.04E-05	1.19E-06
3	2.82E+03	6.81E+01	6.30E-05	2.42E-06	6.08E-02	2.83E-03	4.08E-05	4.12E-07
4	1.77E+03	2.23E+01	9.43E-05	3.70E-06	1.37E-02	6.00E-04	3.39E-05	5.49E-07
5	1.93E+03	2.27E+01	8.48E-05	2.54E-06	8.96E-03	3.07E-04	4.51E-05	6.80E-07
6	2.22E+03	5.42E+01	1.42E-04	5.89E-06	5.20E-03	2.61E-04	1.04E-04	2.72E-06
7	2.30E+03	3.79E+01	1.38E-04	5.47E-06	8.18E-03	3.94E-04	7.86E-05	1.25E-06

**Table. E.1:** **Estimatedkineticsparameters for bivalent analyte model for regenerative titration data sets with standard length of dissociation using BIAevaluation software.** Estimated and standard error (SE) values for kinetics parameters, *k*_*a*1_, *k*_*a*2_, *k*_*d*1_, and *k*_*d*2_, for all 7 data sets.

	Bivalent Analyte Model (BIAevaluation)							
	*k*_*a*1_(*M*^−1^*s*^−1^)		*k*_*a*2_(*RU*^−1^*s*^−1^)		*k*_*d*1_(*s*^−1^)		*k*_*d*2_(*s*^−1^)	
Data set	Estimated	SE	Estimated	SE	Estimated	SE	Estimated	SE
1	5.22E+03	2.59E+02	1.31E-04	6.80E-06	2.30E-01	1.99E-02	7.56E-05	1.86E-06
2	2.17E+03	7.04E+01	1.64E-04	1.60E-05	5.23E-02	7.14E-03	1.02E-04	4.43E-06
3	2.16E+03	4.93E+01	4.29E-05	2.10E-06	2.54E-02	1.28E-03	4.19E-05	3.70E-06
4	2.56E+03	4.78E+01	6.67E-05	3.26E-06	4.66E-02	1.43E-03	1.11E-04	3.53E-06
5	2.50E+03	6.45E+01	6.27E-05	3.13E-06	3.81E-02	2.27E-03	1.59E-04	3.95E-06
6	5.56E+03	1.66E+02	6.63E-06	3.70E-06	6.68E-04	2.59E-05	2.36E-06	1.93E-04
7	5.37E+03	1.24E+02	3.34E-06	2.23E-06	4.38E-04	1.50E-05	1.28E-07	6.02E-05

**Table. E.2:** **Estimated kinetics parameters for bivalent analyte model for regenerative titration data sets with extended length of dissociation using BIAevaluation software.** Estimated and standard error (SE) values for kinetics parameters, *k*_*a*1_, *k*_*a*2_, *k*_*d*1_, and *k*_*d*2_, for all 7 data sets.

	Bivalent Analyte Model (BIAevaluation)							
	*k*_*a*1_(*M*^−1^*s*^−1^)		*k*_*a*2_(*RU*^−1^*s*^−1^)		*k*_*d*1_(*s*^−1^)		*k*_*d*2_(*s*^−1^)	
Data set	Estimated	SE	Estimated	SE	Estimated	SE	Estimated	SE
1	1.89E+03	4.88E+00	5.45E-05	2.96E-07	8.68E-03	2.04E-05	6.00E-05	1.63E-07
2	1.61E+03	1.00E+01	6.43E-05	8.02E-07	5.48E-03	2.27E-04	6.59E-05	1.53E-06
3	2.62E+03	2.39E+01	4.31E-05	1.15E-06	5.23E-02	1.28E-03	5.37E-05	6.42E-07
4	1.87E+03	1.60E+01	5.20E-05	7.97E-07	1.29E-02	2.49E-04	6.98E-05	9.88E-07
5	2.41E+03	2.64E+01	7.22E-05	2.29E-06	3.98E-03	1.27E-04	1.50E-04	3.29E-06
6	6.20E+03	1.16E+02	4.06E-05	1.98E-06	6.13E-04	1.37E-05	7.86E-06	1.43E-05
7	5.75E+03	8.25E+01	1.54E-05	1.10E-06	2.90E-04	5.00E-06	9.47E-06	2.34E-05

## Data Availability

The R script and example data for the bivalent analyte model described in this article are available at https://github.com/DukeCHSI/Bivalent-Analyte-Modeling. The data included in this article are provided as the example data files, and are also available at https://zenodo.org/record/8118397.
